# Cryoconite Hole Location in East-Antarctic Untersee Oasis Shapes Physical and Biological Diversity

**DOI:** 10.3389/fmicb.2020.01165

**Published:** 2020-06-03

**Authors:** Klemens Weisleitner, Alexandra Kristin Perras, Seraphin Hubert Unterberger, Christine Moissl-Eichinger, Dale T. Andersen, Birgit Sattler

**Affiliations:** ^1^Institute of Ecology, University of Innsbruck, Innsbruck, Austria; ^2^Austrian Polar Research Institute, Vienna, Austria; ^3^Center for Medical Research, Medical University of Graz, Graz, Austria; ^4^Unit of Material Technology, University of Innsbruck, Innsbruck, Austria; ^5^SETI Institute, Mountain View, CA, United States

**Keywords:** Anuchin Glacier, 16S rRNA, cryoconite holes, mineralogy, archaea, bacterial activity, biogeochemistry

## Abstract

Antarctic cryoconite holes (CHs) are mostly perennially ice-lidded and sediment-filled depressions that constitute important features on glaciers and ice sheets. Once being hydrologically connected, these microbially dominated mini-ecosystems provide nutrients and biota for downstream environments. For example, the East Antarctic Anuchin Glacier gradually melts into the adjacent perennially ice-covered Lake Untersee, and CH biota from this glacier contribute up to one third of the community composition in benthic microbial mats within the lake. However, biogeochemical features of these CHs and associated spatial patterns across the glacier are still unknown. Here we hypothesized about the CH minerogenic composition between the different sources such as the medial moraine and other zones. Further, we intended to investigate if the depth of the CH mirrors the CH community composition, organic matter (OM) content and would support productivity. In this study we show that both microbial communities and biogeochemical parameters in CHs were significantly different between the zones medial moraine and the glacier terminus. Variations in microbial community composition are the result of factors such as depth, diameter, organic matter, total carbon, particle size, and mineral diversity. More than 90% of all ribosomal sequence variants (RSV) reads were classified as Proteobacteria, Cyanobacteria, Bacteroidetes, Actinobacteria, and Acidobacteria. Archaea were detected in 85% of all samples and exclusively belonged to the classes Halobacteria, Methanomicrobia, and Thermoplasmata. The most abundant genus was *Halorubrum* (Halobacteria) and was identified in nine RSVs. The core microbiome for bacteria comprised 30 RSVs that were affiliated with Cyanobacteria, Bacteroidetes, Actinobacteria, and Proteobacteria. The archaeal fraction of the core microbiome consisted of three RSVs belonging to unknown genera of Methanomicrobiales and Thermoplasmatales and the genus *Rice_Cluster_I* (Methanocellales). Further, mean bacterial carbon production in cryoconite was exceptionally low and similar rates have not been reported elsewhere. However, bacterial carbon production insignificantly trended toward higher rates in shallow CHs and did not seem to be supported by accumulation of OM and nutrients, respectively, in deeper holes. OM fractions were significantly different between shallower CHs along the medial moraine and deeper CHs at the glacier terminus. Overall, our findings suggest that wind-blown material originating south and southeast of the Anuchin Glacier and deposits from a nunatak are assumed to be local inoculation sources. High sequence similarities between samples from the Untersee Oasis and other Antarctic sites further indicate long-range atmospheric transport mechanisms that complement local inoculation sources.

## Introduction

One typical characteristic of glaciers and ice sheets is the occurrence of cryoconite holes (CHs). These small water bodies form when dark organic and inorganic debris attach to ice surfaces and consequently decrease the albedo locally (Anesio et al., 2009). Solar irradiation promotes melting of the matter into deeper ice layers until the rates of downward movement and surface ice ablation enter a steady state equilibrium (Gribbon, 1979; Hodson et al., 2013). The basins are filled with sediments in the bottom and liquid water on the top (Takeuchi et al., 2001).

In the late 19th century, A. E. Nordenskiöld recognized the ecological importance of these miniature lakes on the Greenland ice sheet (Leslie, 2011). About one hundred years later, CH research was pioneered in Antarctica by Wharton et al. (1981) however, despite the gained knowledge and recent technological advances, they remain a “dark biological secret of the cryosphere” (Cook et al., 2016).

CHs are currently perceived as microbial hotspots (Edwards et al., 2013; Weisleitner et al., 2019a) because their biodiversity and productivity are much higher than in other supraglacial zones (Anesio et al., 2009; Cook et al., 2016). The presence of viruses, bacteria, micro-algae, fungi, protozoa, and sometimes metazoans indicate that CHs are simple trophic systems (Mueller et al., 2001; Mueller and Pollard, 2004; Porazinska et al., 2004; Hodson et al., 2008; Sommers et al., 2019). Primary producers such as cyanobacteria fix inorganic carbon via photosynthesis and provide nutrients for the heterotrophic fraction of the community (Mueller et al., 2001; Morgan-Kiss et al., 2006; Bagshaw et al., 2016). Production and respiration rates are mainly controlled by light availability and temperature regime and hence changes seasonally (Bagshaw et al., 2016). During austral summer, primary production can exceed respiration and liquid water may persist for up to 10 weeks in Antarctic CHs. This effect may be reversed during the dark season (Fountain et al., 2004; Bagshaw et al., 2007).

Biological processes within CHs play an important role in both local and downstream environments (Anesio et al., 2010). For example the build-up of dark organic matter (OM) within CHs enhances local supraglacial melt rates (Takeuchi et al., 2001) and hence increases the water availability within CHs. Further, in comparison to hydrologically connected CHs, isolated ones promote recycling and production of nutrients and hence support downstream ecosystems once being re-connected to a hydrological system (Bagshaw et al., 2007; Samui et al., 2018). Such a nutrient flush can be caused by a warming event as recorded in the McMurdo Dry Valleys (MDVs) that led to a temporarily increased primary production in Lake Fryxell (Bagshaw et al., 2013). Further, CH bacterial communities from the Anuchin Glacier damming the perennially ice-covered Lake Untersee (East-Antarctica) were identified as important microbial source inoculum for benthic microbial communities that occur in the lake (Weisleitner et al., 2019b). However, the question is still open how relevant those supraglacial communities are for the local carbon production. Antarctic productivity within CHs is generally extremely low and is hampered by harsh conditions (Foreman et al., 2007).

Compared to CHs from other regions such as the Alps or the High Arctic, where the limiting factor of a permanent ice cover of CHs is mostly missing, those in Antarctica often form perennial ice-lids and therefore attenuate most of the incoming photosynthetically active irradiation (PAR) before reaching the bottom of the CHs (Fountain et al., 2004). Also, Antarctic conditions such as low temperatures and strong katabatic winds reduce the occurrence of surface waters and therefore may increase the longevity of Antarctic CHs compared to their counterparts from other glaciated regions which may be flushed more frequently (Hodson et al., 2008) as a result of melt. Measurements of excess Cl^–^ in ice-lidded CHs from the Canada glacier suggest these habitats persist for up to 5 years (Bagshaw et al., 2007). In contrast, the first reported Antarctic radiocarbon data from CHs indicate that they may occur in isolation for even thousands of years (Lutz et al., 2019) and could provide long-term refugia for unfavorable conditions which were found during snowball earth (Vincent et al., 2000). Isolation also prevents further inoculation by bioaerosols once the lid is formed. Although being separated from the atmosphere, microbial communities in spatially close CHs are more similar to each other compared to those that are separated at a larger scale (Darcy et al., 2018), indicating that local sources lead to the formation of CHs and hence should be considered as an important aspect in the colonization of CHs (Webster-Brown et al., 2015).

In contrast to Alpine and Arctic regions the number of Antarctic CH studies is low and mostly limited to glaciers around the MDVs (e.g., Christner et al., 2003; Fountain et al., 2004, 2008; Porazinska et al., 2004; Fortner et al., 2005; Bagshaw et al., 2007, 2016; Foreman et al., 2007; Telling et al., 2014; Webster-Brown et al., 2015; MacDonell et al., 2016) with some geographical exceptions (e.g., Cameron et al., 2012; Hodson et al., 2013; Obbels et al., 2016; Zdanowski et al., 2017). Hence, compared to the “prime study sites” such as the MDVs, little is known about the microbial ecology in CHs from e.g., Queen Maud Land (East-Antarctica). There, only a handful of studies were conducted (e.g., Obbels et al., 2016; Samui et al., 2018; Lutz et al., 2019; Weisleitner et al., 2019b) although this pristine area exceeds the size of Greenland and hence is under-represented on a geographical scale. One of the main study areas in Queen Maud Land is Lake Untersee with the adjacent Anuchin glacier from which microbial diversity and various inoculation vectors have been investigated, however, there is no information about activity within their ice-sealed environments.

For the past 10 years, the Anuchin Glacier in the Lake Untersee Oasis has been serving as a model habitat for these environments to shed light on microbial and biogeochemical processes outside the core location near McMurdo. CHs from glaciers have been identified as important source inoculum for the adjacent perennially ice-covered lakes as it has been shown for Anuchin Glacier and its role for Lake Untersee (Weisleitner et al., 2019b). Very little is known about the minerogenic composition and its role as nutrient source for microbial communities, or how active microbial communities can be under the peculiarity of permanently ice-covered CHs. Which role can we attribute bacteria in terms of carbon producers? In this study we further characterized these CHs by determining its archaeal and bacterial diversity and defined a core microbiome. Further, we explored local temperature variations within the ice matrix as proxy for cryoconite temperature conditions. We hypothesized (1) that the CH minerogenic composition differed between the medial moraine and those from other zones, and (2) that the CH community composition, OM content and productivity are mirrored by the depth of the CH due to possible accumulation.

## Study Site

**FIGURE 1:**
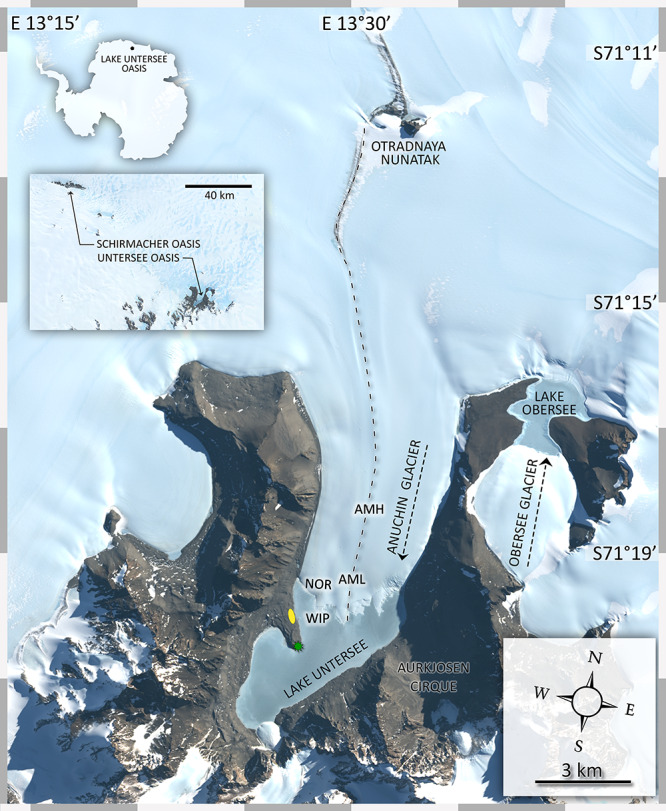
Map of the study site. The insert map indicates the position of the study site in context with the Schirmacher Oasis. Lake Untersee (610 m a.s.l.) and Lake Obersee (805 m a.s.l.) are partly connected by the Anuchin Glacier. The position of the sampling sites for CHs (zone “medial moraine”: AMH, AML, zone “glacier terminus”: ANR, WIP) are indicated in the map. The green star shows the location of a meteorological station and the yellow ellipse depicts the position of the campsite during the expedition. The dashed line along the glacier indicates the position of the medial moraine. The flow directions of the Anuchin Glacier and the “Obersee Glacier” are indicated by dashed arrows. The image was captured by the Landsat 8 satellite on 09 MARCH 2019 (download via Earth Explorer by United States Geographical Survey). The true color image (4-3-2 RGB, 30 m px^– 1^) was combined with the high-resolution band 8 (grayscale, 15 m px^–1^).

The north-south orientated Anuchin Glacier is located in the Gruber Mountains (71.348°S, 13.517°E, Figure 1) and formed after the last glacial maximum (Schwab, 1998). It extends over an area of ∼34 km^2^ (length ∼8 km, width 4.2 ± 1.2 km). A geodetic survey (Wand and Perlt, 1999) and interferometric synthetic-aperture measurements (Rignot et al., 2014) indicate that the glacier surface speed is ∼9 m a^–1^. A similar flow speed (8.4 m a^–1^) was recently reported by Faucher et al. (2019). The elevation difference between 8 km up-glacier and the glacier terminus is 218 m, corresponding to an average slope angle of 1.56° along the medial moraine (REMA dataset, Howat et al., 2018). The Anuchin Glacier dams the perennially ice-covered Lake Untersee at its northern end which is known for its modern, large conical stromatolites and its anoxic sub-basin with exceptional high methane production rates (Wand et al., 2006; Andersen et al., 2011). Supraglacial melt does not provide water for recharge but subaqueous glacier melt contributes 40–45% to the annual lake water budget which is likely complemented by subglacial inputs (Faucher et al., 2019). Lake Untersee has no outlet and hence water loss occurs by ablation of the ice cover (Hermichen et al., 1985). Here, the dominating ablation process is sublimation as a result of low summer temperatures and strong katabatic winds, reaching up to 40–75 cm a^–1^ (Andersen et al., 2015; Faucher et al., 2019). Similar ablation rates (50–60 cm a^–1^) were measured at the lower part of the Anuchin Glacier (Wand et al., 2006). However, rates within the blue ice zone between the nearby Schirmacher and Untersee Oasis are significantly lower (5–15 cm a^–1^, Scheinert et al., 2006), indicating that local meteorological conditions within the oasis promote ablation.

## Materials and Methods

### Sampling

**FIGURE 2:**
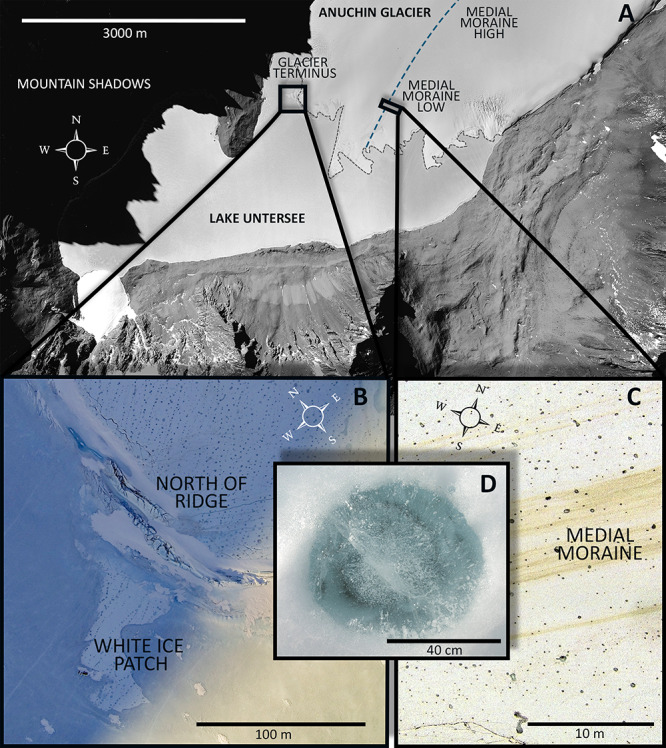
**(A)** Aerial image of Lake Untersee and the lower part of the Anuchin Glacier (Bundesamt für Kartographie und Geodäsie, Frankfurt am Main, Germany). The medial moraine is indicated by a dashed blue line. The glacier-lake interface is indicated by a gray dashed line. **(B)** Aerial overview of the zone “glacier terminus.” **(C)** Level and contrast enhanced aerial image showing a representative site along the medial moraine. **(D)** Typical morphology of an ice-lidded cryoconite hole from the Anuchin Glacier. Note that the orientations in Figures 2A–C differ from each other.

During November and December 2015 fourteen CHs were collected across the Anuchin Glacier. Four sites were grouped into two zones, namely “medial moraine” and “glacier terminus” (Figure 2A). The first zone “glacier terminus” (Figure 2B) included a site north of an ice ridge that separated the glacier ice from the lake ice (Anuchin North of Ridge, ANR) and the white ice patch that appears to be glacial ice embedded in the lake ice cover of Lake Untersee (White Ice Patch, WIP). The second zone was located further up-glacier along the medial moraine (Anuchin Moraine High, AMH) and at the lower part of the medial moraine (Anuchin Moraine Low, AML). At least 3 CHs were collected at each site. Representative medial moraine sites and a typical CH are shown in Figures 2C,D, respectively.

Depending on the depth of the CHs, samples were either collected with an ethanol-rinsed Kovacs ice corer (Mark III, 7.25 cm diameter) in combination with a Boschhammer GBH 36V-LI PLUS electrical drill or with a sterile spatula after removal of surface ice using a sterilized ice ax or chisel. For each hole, GPS location, diameter and depth were recorded. All samples were stored in sterile polyethylene bags and kept frozen until the end of the expedition and then shipped via Cape Town to the University of Innsbruck and subsequently stored at −20°C until further use.

### Temperature Depth Profile Time Series

To study differences in the temperature regime of CHs, three sites that represented the medial moraine (AML/AMH), ANR and WIP were chosen. In contrast to ice at the medial moraine that was dark-colored and interspersed with particulates (Figure 2C), ice at the site ANR appeared white and macroscopically free of particles. Despite being embedded within the ice cover of Lake Untersee, the site WIP appeared like ice from the site ANR.

At each site, a 1 m deep hole was drilled and three Tidbit temperature loggers (accuracy ± 0.21°C from 0 to 50°C) were attached to a line and then placed into the hole at an initial depth of 1 m, 0.5 m and just below the surface (referred to as 0 m), respectively. The holes were re-filled with small pieces of ice once the loggers were put in place. Sensor readings were logged with an interval of 15 min and covered a period of 11 days (28.11.2015 – 08.12.2015. To ensure that the temperature loggers being used did not inadvertently skew the *in situ* cryoconite readings, the albedo was compared between the loggers and other CHs with a custom-built device utilizing an Ibsen OEM FHT-315 spectrometer. Milled samples were illuminated with a white LED and a tungsten filament and spectral reflectances of cryoconite and temperature loggers were recorded in a range of 470–1100 nm under identical light conditions. To allow a direct comparison, the integration time was adjusted to the brightest measured sample and then left unchanged for all other samples (Supplementary Figure 1). Further, solar radiation, soil temperatures (sensors buried at 0.01, 0.10, 0.22 m) and air temperature records from an automatic weather station operating at the shore of Lake Untersee (location shown in Figure 1) has been used (Andersen et al., 2015). Descriptive statistics were calculated with the R packages“Psych” (Revelle, 2019) and permutation distribution clustering was performed with the R package “PDC” (Brandmaier, 2015). Heatmaps were created with the R package ggplot2 (Wickham, 2011).

### Particle Size

The following analyses including sub header 3.5 were done on sediments collected from CHs. Gravimetric particle size distribution was determined by sieving into five fractions (>400 μm, 100–400 μm, 40–100 μm, 10–40 μm, and <10 μm). Large fractions were dry sieved, and the two smallest fractions were wet sieved. Next, the samples were dried in pre-weighted containers at 40°C before determining the dry weight. The respective particle size distribution was expressed as the relative mass proportion per sample. The particle size distributions were visualized in a ternary plot using the R package “ggtern” (Hamilton and Ferry, 2018).

### X-Ray Diffraction (XRD) and Thermogravimetric Analysis (TGA)

Samples for X-ray diffraction and thermogravimetric analysis were dried at 70°C for 48 h and subsequently milled with a Retsch MS 1100 high-speed micromill equipped with an agate-beaker for 3 min.

#### Powder X-ray Diffraction (XRD)

The mineralogical composition in the homogenous powder was analyzed with a Panalytical Empyrean XRD diffractometer equipped with a monochromator and a 1D Pixel detector. XRD measurements were carried out with Cu Kα radiation operating at 40 kV and 40 mA. Spectra were collected over a 2θ range of 5–70° in 0.013 steps with a scan speed of 0.16° s^–1^ resulting in a total scan time of 7 min. All samples were measured using a standard sample holder except samples having insufficient mass (CRY1, CRY2) were measured using a silicon crystal sample holder. Spectra were analyzed using a Bruker DiffracEVA software by comparing with PDF2 patterns.

#### Thermogravimetric Analysis (TGA)

The total amount of OM was estimated by thermogravimetric analysis. Between 100 to 150 mg of dried and milled cryoconite sediment samples were loaded into a Netzsch STA 449. Weight loss was recorded continuously while samples were heated at 10 K min^–1^ from 25°C up to 1000°C. Mass loss was calculated for the following temperature intervals: 38–105°C, 105–200°C, 200–350°C, 350–520°C, and 520–1000°C. STGA analysis enabled a differentiation between thermo-labile (200–350°C) and thermo-stable (350–520°C) OM. This classification was also used by Smith et al. (2016) for Antarctic CHs in the MDVs. The detection limit was set to Δ0.1% which corresponded to Δ10–15 μg OM – depending on the initial mass load in the thermal analyzer. This threshold was well above the measurement precision of the instrument (0.1 μg).

### Total Carbon, Nitrogen, and Phosphorus

For total carbon and nitrogen analysis 50 mg of milled samples were transferred into tin capsules and combusted in a Nitrogen and Carbon Analyzer in the NC soils configuration (Flash EA 1112, Thermofisher Scientific). Acetanilide was used for the calibration line. The percentage of carbon and nitrogen was then referred to the sample mass as total C (mg g^–1^) and total N (mg g^–1^).

Total phosphorus was quantified with the molybdate method after Vogler (1966). Briefly, pre-weighed and pulverized sediments were suspended in deionized water, sulfuric acid and then heated to 160°C for 12 h. Next, the samples were oxidized with hydrogen peroxide, diluted with distilled water and cooled down to room temperature. Then, ascorbic acid (10%) and sodium hydroxide (20%) were added. After neutralizing the samples with Vogler solution, the absorbance features were measured against a H_3_PO_4_ standard (Tritisol, Merck, Darmstadt, Germany) at 885 nm with a U-2001 spectrophotometer (Hitachi, Tokyo, Japan).

### Scanning Electron Microscopy (SEM)

For representative SEM images, 250 μL of dislodged cell-extract (Duhamel and Jacquet, 2006) and untreated samples were filtered onto 0.2 μm polycarbonate filters (Osmonics, DK) and dried overnight at 40°C. Samples were sputtered with tungsten and subsequent SEM images were recorded with a Quanta 3D 200 Double Beam SEM microscope (FEI, Eindhoven, Netherlands) using 20,000x magnification and 0.3 mbar at an acceleration voltage of 5.0 kV.

### Microbial Abundance

Melted cryoconite was subsampled and weighed. Dry weights were calculated from aliquots after water removal at 70°C for 48 h. Next, cells were dislodged according to the protocol of Duhamel and Jacquet (2006) which is based on both; chemical (tween 80, pyrophosphate, formaline) and physical (sonic bath, pre-filtration and centrifugation) treatment. Then, 250 μl aliquots from the cell suspensions were diluted with 750 μl MilliQ water, stained with DAPI (4’,6-Diamidine-2-phenylindol) at a final concentration of 0.2% v/v (Porter and Feig, 1980) and then filtered onto 0.2 μm polycarbonate filters (Osmonics, DK). Images were recorded with an Axiocam HRc attached to a Carl Zeiss Imager Z1 microscope using a 63x oil lens. For each sample aliquot, 40 randomly selected areas were photographed using a 365 nm excitation source and the filter set EX G 365, BS FT 395, EM BP 445/50. Cells were enumerated with the cell counter plugin within the imaging analysis software Fiji (Schindelin et al., 2012).

### Bacterial Activity

Bacterial activity was estimated by the incorporation of ^3^H-leucine by the micro-centrifuge method (Kirchman, 2001). Incubation for all samples took place under simulated *in situ* temperature conditions (0.1°C) in HAAKE water baths. ^3^H-leucine was added with a final concentration of 40 nM to sample triplicates with ca. 1.5 mL of a mixture of sediment and water and two formalin-killed control samples. After 72 h, the incubation was terminated with 90 μL of 100% trichloroacetic acid (TCA). Next, the samples were centrifuged at 16,000 *g* for 10 min and then washed, centrifuged again and the supernatant was aspirated with 5% TCA and 80% EtOH. The final supernatant was aspirated and the remaining sediment weighted for the calculation of bacterial production. The samples were measured in a liquid scintillation counter (Beckman LSC 6000 IC) after adding 1 mL of scintillation cocktail (Beckman Ready Safe).

### Microbial Community Composition and Statistics

#### Sample Processing and Next Generation Sequence Analysis

Cryoconite samples were carefully thawed and DNA was extracted with a PowerSoil DNA isolation kit. The 16S rRNA gene amplicons were generated with Illumina-tagged universal primers F515 and R806 as proposed by the Human Microbiome Project (Caporaso et al., 2012). For Archaea, primers 344f and 915r (Klindworth et al., 2013) in combination with a subsequent nested PCR approach with the primer pair S-D-Arch-0519-a-S-15/S-D-Bact-0785-b-A-18 were used (Koskinen et al., 2017). Next, library preparation and MiSeq sequencing were performed (Klymiuk et al., 2016). The R package DADA2 was used to process and filter the raw sequences according to the proposed pipeline (Callahan et al., 2016) and the taxonomy was assigned to the SILVA database (Quast et al., 2013). This procedure resulted in a Ribosomal Sequence Variants table (RSV). A more detailed workflow covering all steps from sample processing to NGS analysis and the use of negative controls is outlined in Weisleitner et al. (2019b) and references therein. Sequence accession numbers are listed in Supplementary Table 1.

#### Statistical Analysis

Based on the RSV table, alpha and beta diversity were calculated using the R package Phyloseq (ver 1.20.0) (McMurdie and Holmes, 2013). To compare the single sites (AML, AMH, ANR, WIP) and the zones (medial moraine, glacier terminus), Shannon, Observed and Inverse Simpson indices were compared. As previously discussed, counts were not rarefied (McMurdie and Holmes, 2014; Weisleitner et al., 2019b). The data distribution was tested with a Shapiro–Wilk test and depending on the outcome, significant differences were either tested with a Kruskal–Wallis test (for not normally distributed data) or with an Analysis of Variance (ANOVA, for normally distributed data). Dunn’s test with Bonferroni correction was used as *post hoc* test. We also tested for significantly different abundant taxa between samples from the medial moraine and outside the medial moraine following the R package DESEQ 2 (v.1.20.0) pipeline (Love et al., 2014). Differences in microbial communities between sites were visualized with a Principal Coordinates Analysis (PCoA, R package Phyloseq). Analysis of similarities (ANOSIM) based on a Bray–Curtis RSV dissimilarity matrix and PERMANOVA were calculated with the R package vegan (Oksanen et al., 2007).

The core microbiome was defined as RSVs that had a larger or equal average relative abundance of 0.1% across all sites (AMH, AML, ANR, WIP) - but not necessarily within all single samples. To put the core microbiome into context with other RSVs, the mean relative abundances across all sites of the core microbiome and RSVs that occurred at less sites with a mean relative abundance ≥0.1% were plotted in a sunburst diagram using the browser-based tool RawGraphs (Mauri et al., 2017).

For the metadata set, residual diagnostics were performed for all variables. Those that did not meet the criteria for analysis of variance (ANOVA) were log10 transformed and re-tested. ANOVA and Tukey’s *post hoc* test were then used to test for significant differences between all four sites (AML, AMH, WIP, ANR). Differences between the zones medial moraine (AML, AMH) and the glacier terminus (ANR, WIP) were compared with Welch’s *t*-test.

We used non-parametric statistical tests for log10 transformed variables that did not meet all criteria for the above-mentioned statistical approaches (TC, mineral diversity, Kruskal–Wallis test – instead of ANOVA or Mann–Whitney test – instead of Welch’s *t*-test). Further, a principal component analysis (PCA) of all variables was computed to explain variations between sites.

## Results

### Mineral Composition and Particle Size

**FIGURE 3:**
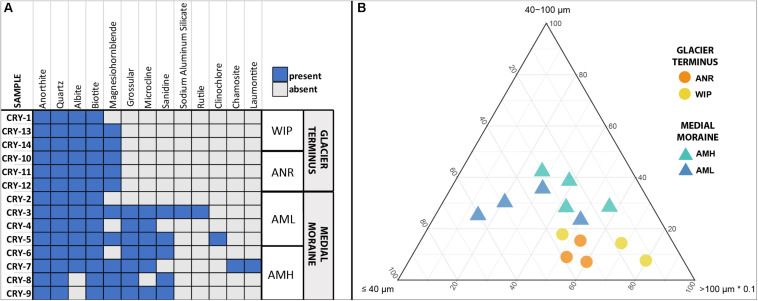
**(A)** Site occurrences of minerals based on XRD measurements. **(B)** Ternary plot of particle sizes. Size classes were combined to capture all data within one plot (x ≤ 10 μm + 10 – 40 μm, *y* = 40 – 100 μm, *z* = 100 – 400 μm + >400 μm). Note that the *z*-axis is scaled with factor 0.1.

In total 13 different minerals were identified (Figure 3A). The mineral diversity differed significantly between zones (Mann–Whitney *U* test, *W* = 5.5, *p*-value = 0.01696) and generally decreased with depth (Pearson correlation, adj. *r*^2^ = 0.36, *p* = 0.001). The average mineral diversity was higher along the medial moraine (AMH: 7.25 ± 1.26, AML: 7.25 ± 2.75) than at the glacier terminus (ANR: 5 ± 0, WIP: 4.67 ± 0.58) and inferred that also the mineral composition differed between samples collected along the medial moraine (AMH, AML) and those from regions closer to Lake Untersee (ANR, WIP). Albite, Anorthite, Biotite, and Quartz were found in all samples except C8 and C9 which lacked Albite. Samples C1 and C2 harbored only four different minerals while C3 contained 10 different minerals. Sanidine, Grossular, Microcline, Sodium Aluminum Silicate, Rutile, Clinochlore, Chamosite, and Laumontite absent at the glacier terminus (Figure 3A).

Particle sizes were classified into five groups (>10 μm, 10–40 μm, 40–100 μm, 100–400 μm, >400 μm) and varied across the sites. CHs along the medial moraine had a slightly higher frequency of small particles compared to those from the glacier terminus (Figure 3B). The particle fraction 40–100 μm differed significantly between ANR-AMH, ANR-AML and WIP-AML [ANOVA, *F*(3,10) = 7.047, *p* = 0.008, Tukey’s *post hoc* test] and the ratio of combined particle sizes < 100 μm and >100 μm increased with CH depth (Pearson correlation, adj. *r*^2^ = 0.37, *p* = 0.012). The complete particle size distribution is shown in Supplementary Figure 2 and representative SEM images of minerals are depicted in Supplementary Figure 3.

### Proxy for Cryoconite Hole *in situ* Temperatures

The coldest overall mean CH proxy temperatures across all depths were recorded at the Anuchin Glacier (clean ice: −2.19 ± 4.73°C, medial moraine: −2.02 ± 3.12°C) while WIP was slightly warmer (−1.7 ± 3.14°C). Mean temperatures and temperature fluctuations across all sites decreased with depth (surface: 0.69 ± 5.24°C, middle: −2.51 ± 1.29°C, bottom: −4.09 ± 1.03°C). Further, the strongest temperature decrease with depth was at the clean ice (0.63°C 10 cm^–1^) followed by the medial moraine (0.50°C 10 cm^–1^) and the white ice patch (0.30°C 10 cm^–1^). Further, Pearson correlation coefficients calculated from ambient air temperature (−1.9 ± 2.64°C) and the temperature loggers indicated that temperature dynamics at the medial moraine were more de-coupled from the air temperature (*r*^2^ = 0.64) compared to the WIP (*r*^2^ = 0.68) and clean ice site (*r*^2^ = 0.79). This was confirmed by permutation distribution clustering (Supplementary Figure 4) which showed that air temperature and surface ice temperatures at the glacier were more similar than the surface temperature of the white ice patch despite being geographically closer to the automatic weather station that recorded ambient air temperatures 2 m above lake level and soil temperatures at various depths (Andersen et al., 2015).

**FIGURE 4:**
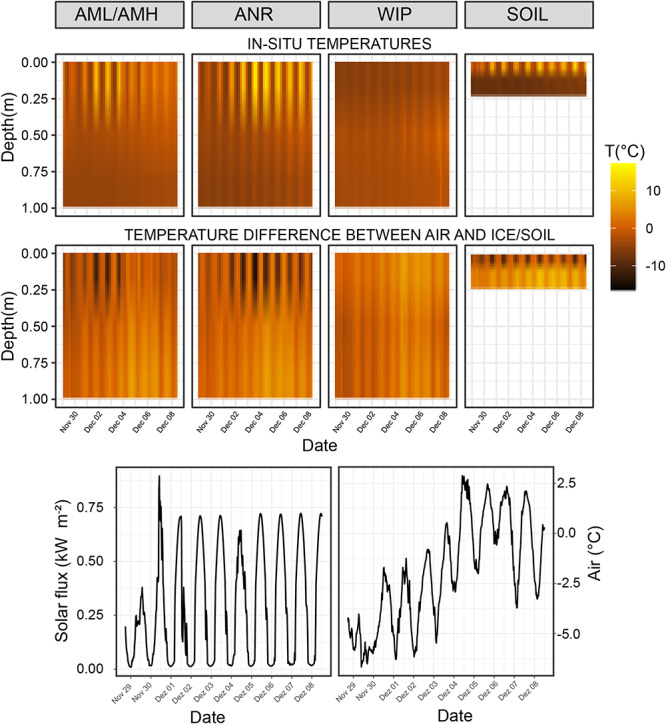
**Top row:** Temperature time series of interpolated depth gradient. Temperature loggers were installed at 0, 0.5, and 1.0 m deep in the ice covers, respectively. Temperature probes for the soil time series were buried at 0.01, 0.1, and 0.22 m depth. **Bottom row:** Temperature differences between air temperature and CH/soil.

The largest temperature difference between the surface and 1 m below the ice was recorded in clear ice (22.34°C). Average soil temperature differences between 0.01, 0.1, and 0.22 m depth were 9.51 and 9.91°C, respectively. Negative temperature differences (i.e., warmer ice at 1 m depth compared to the surface ice) were recorded at all sites except for the soils and the maximum was reached during the night at the WIP site (−3.55°C, Figure 4 – top row). For sites at the glacier, negative differences between ice and air temperatures were observed more frequently at the ice surface while this was more common across all depths at the site WIP (Figure 4 – bottom row).

### Physical and Biogeochemical Characteristics

**FIGURE 5:**
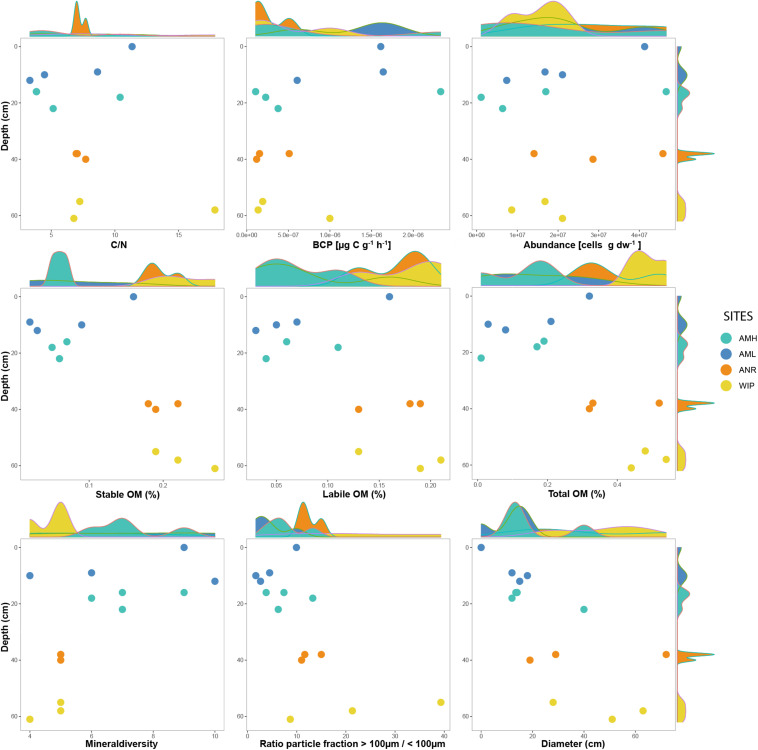
Selected variables plotted along against depth. Marginal plots indicate the sample distribution for each variable.

CH depths ranged from 0 to 62 cm and were on average 3.75 times deeper at the glacier terminus than those along the medial moraine [ANOVA, *F*(3,10) = 130.9, *p* ≤ 0.001]. The sites within these zones differed by a factor 2.2 for the medial moraine (AML vs. AMH) and by 1.5 for the glacier terminus (ANR vs. WIP) and at least one sample was located every 10 cm across the full depth range (Figure 5). Deeper CH layers also trended toward larger CH diameter (Pearson correlation adj. *r*^2^ = 0.5, *p* = 0.029) and were significantly different between the zones (Welch test: *t* = −2.925, df = 7.0437, *p*-value = 0.02203).

**FIGURE 6:**
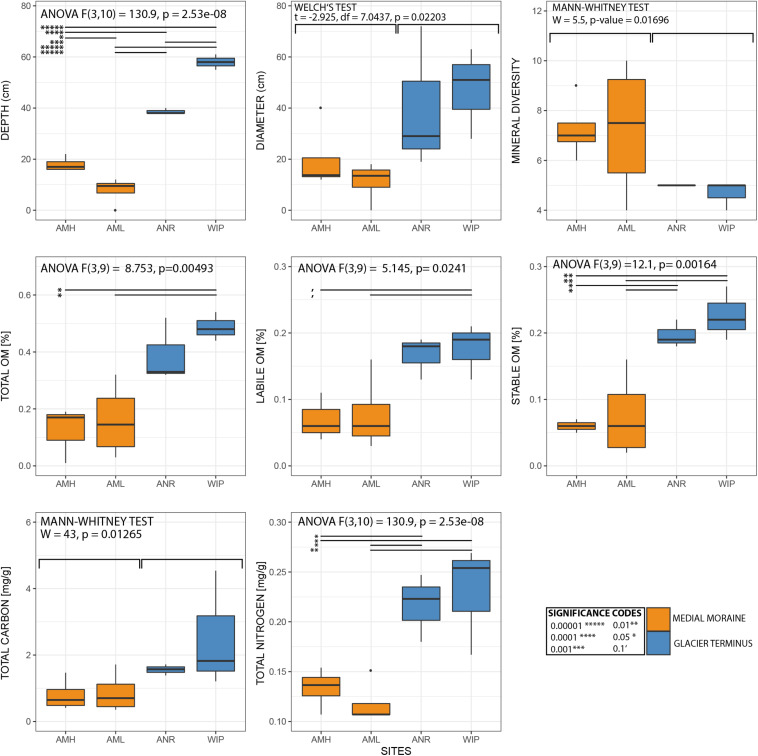
Boxplots of selected variables. Note that sample CRY-7 was identified as outlier and hence was excluded from the boxplots and statistical analysis for organic matter.

Total phosphorus contents in cryoconite were similar across all sites (0.51 – 0.53 mg g^–1^) and C/N ratios were slightly higher at the glacier terminus than at the medial moraine (8.93 ± 4.39 and 6.37 ± 3.24, respectively). More pronounced differences [*F*(3,10) = 10.12, *p* = 0.00225] were observed for total nitrogen that ranged on average per site from 0.07 ± 0.03 mg g^–1^ at AML to 0.23 ± 0.05 mg g^–1^ at WIP. Also, total carbon contents were higher at the glacier terminus (Mann–Whitney *U* test, *W* = 43, *p*-value = 0.01265) and varied between 0.58 ± 0.30 mg g^–1^ at AML and 2.52 ± 1.77 mg g^–1^ at the site WIP (Figure 6).

Bacterial carbon production (BCP) was exceptionally low at all sites and ranged between 1.07^∗^10^–07^ and 1.49^∗^10^–05^ μg C g dw^–1^ h^–1^ (AMH: 1.28^∗^10^–06^ ± 4.80^∗^10^–07^ μg C g dw^–1^ h^–1^, AML: 7.61^∗^10^–07^ ± 9.12^∗^10^–07^ μg C g dw^–1^ h^–1^, ANR: 2.61^∗^10^–07^ ± 1.77^∗^10^–07^ μg C g dw^–1^ h^–1^, WIP: 4.43^∗^10^–07^ ± 0.94^∗^10^–07^ μg C g dw^–1^ h^–1^). CRY2 was not within the interquartile range as the BCP rate was two orders of magnitudes higher than most other samples. BCP generally decreased with depth (Pearson correlation, adj. *r*^2^ = 0.15, *p* = 0.1106) and was not significantly different between sites (Figure 6).

Further, labile [ANOVA, *F*(3,9) = 5.145, *p* = 0.0241] and stable OM contents [ANOVA, *F*(3,9) = 12.1, *p* = 0.00164] were higher at the glacier terminus and correlated positively with depth (labile OM: adj. *r*^2^ = 0.6, *p* = 0.001, stable OM: adj. *r*^2^ = 0.4, *p* = 0.012). Consequently, total OM (sum of labile and stable OM fractions) showed the same trends (Supplementary Figure 5). In contrast, mean cell abundances were in the same order of magnitude at the medial moraine (1.97^∗^10^7^± 1.64 ^∗^10^7^ cells g dw ^–1^) and the glacier terminus (2.25^∗^10^7^± 1.32^∗^10^7^ cells g dw ^–1^) and did not follow any strong pattern along the depth gradient.

Descriptive statistics of all variables are listed in Table 1 and selected variables plotted along a depth gradient are shown in Figure 5. Further, boxplots from most variables and corresponding statistical analyses are depicted in Figure 6.

**TABLE 1:** Descriptive statistics of the metadata set (mean ± 1 sd).

	**Medial moraine**		**Glacier terminus**	
	**AMH (*n* = 4)**	**AML (*n* = 4)**	**ANR (*n* = 3)**	**WIP (*n* = 3)**
Depth (cm)	18(±2.83)	7.75(±5.32)	38.67(±1.15)	58(±3)
Diameter (cm)	19.88(±13.44)	15(±3)	40(±28.16)	47.34(±17.79)
TC (mg g^–1^)	0.8(±0.48)	0.58(±0.3)	1.56(±0.17)	2.52(±1.77)
TN (mg g^–1^)	0.12(±0.05)	0.07(±0.03)	0.22(±0.04)	0.23(±0.05)
TP (mg g^–1^)	0.052(±0.001)	0.053(±0.001)	0.051(±0)	0.052(±0.002)
C/N	6.97(±2.95)	8.32(±1.3)	7.25(±0.43)	10.67(±6.49)
Total OM (%)	0.78(±1.32)	0.1(±0.03)	0.38(±0.19)	0.45(±0.05)
Stable OM (%)	0.06(±0.01)	0.08(±0.06)	0.2(±0.02)	0.23(±0.04)
Labile OM (%)	0.07(±0.04)	0.08(±0.06)	0.17(±0.03)	0.18(±0.04)
Mineral diversity	7.25(±1.26)	6.67(±3.06)	5(±0)	4.67(±0.58)
Cell numbers (g dw^–1^)	5.14*10^07^(±6.39*10^07^)	6.65*10^07^(±6.90*10^07^)	1.25*10^08^(±1.10*10^08^)	6.83*10^07^(±4.28*10^07^)
BCP (μg C dw^–1^ h^–1^)	4.43*10^−07^(±3.94*10^−07^)	4.68*10^−06^(±5.89*10^−06^)	7.61*10^−07^(±9.12*10^07^)	2.61*10^−07^(±1.77*10^−07^)

*Sample CRY-7 (AMH) was excluded from the organic matter dataset because the mineral laumontite likely interfered with the TGA analysis (see full dataset in Supplementary Figure 5).*

**FIGURE 7:**
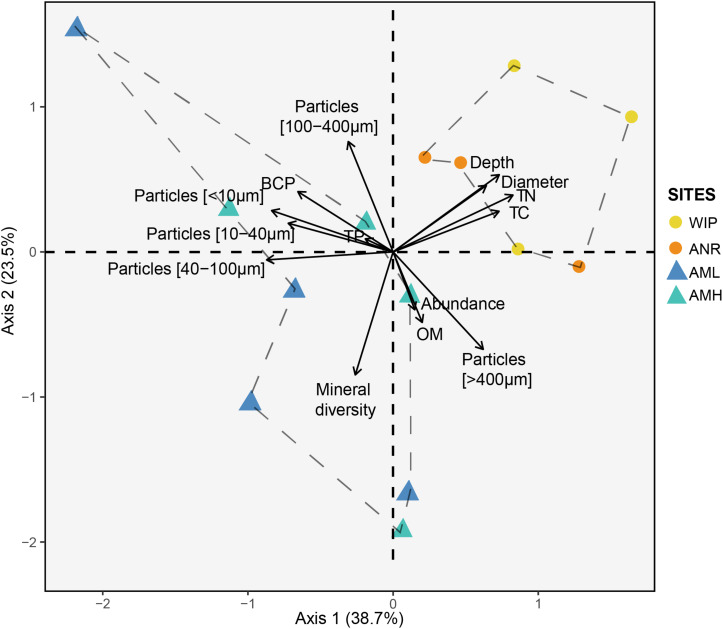
Principal component analysis of the CH dataset (excluding 16S rRNA amplicon data). Circles and triangles represent samples from the glacier terminus and the medial moraine, respectively. Dashed lines serve as a visual guidance.

Next, we performed a PCA to define dissimilarities between sites and variables that accounted for these differences (Figure 7). The first two PCA axes explained 62.2% of the data variability. CHs from the glacier terminus clustered separately and were more similar to each other than those from the medial moraine. The zone glacier terminus was positively correlated with depth, diameter, total nitrogen, and total carbon.

### Microbial Community Composition

#### Bacteria

**FIGURE 8:**
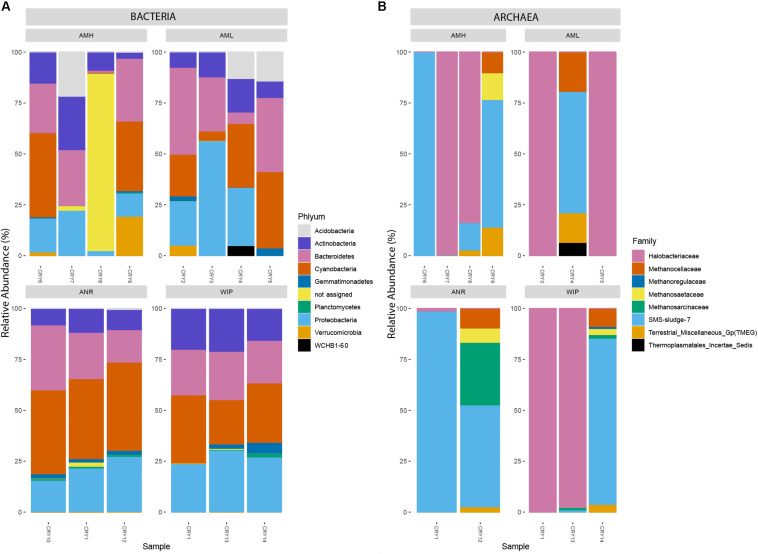
**(A)** Relative abundance of the 100 most abundant RSVs categorized by their respective phyla. **(B)** Relative abundance of all archaea-related RSVs at family level. The upper two bar charts represent samples from the medial moraine and the lower bar charts depict samples from the glacier terminus.

Amplicon sequencing for bacteria resulted in a total of 271,152 read counts ranging from 3,545 (CRY5) to 27,714 (CRY4) resulting in 559 taxonomic observations within 14 samples. CHs were dominated by Proteobacteria (26.54%, 197 RSVs), Cyanobacteria (25.45%, 25 RSVs), Bacteroidetes (23.49%, 118 RSVs), Actinobacteria (13.38%, 48 RSVs), and Acidobacteria (3.20%, 19 RSVs) which made up 92% of all counts. 12.05 and 1.98% of RSVs could not be assigned to a known genus or phylum, respectively. The two most abundant RSVs that were assigned on genus level belonged to the phylum Cyanobacteria (*Tychonema* 10.04%, *Chamaesiphon* 9.82%). The 100 most abundant bacteria related RSVs are depicted in Figure 8A.

#### Archaea

Archaea have been identified with the nested PCR approach in all CHs except in samples (CRY2, CRY10). Within the 12 remaining samples 88,149 read counts allocated to 24 RSVs and 14 of them were affiliated with the order Halobacteriales (89.1% of all counts). Members of Methanomicrobiales accounted for 10.01% of the reads and were affiliated to five RSVs. Combining counts from the remaining five sequences made up less than one percent and belonged to Methanocellales, Methanosarcinales, Thermoplasmatales. One RSV belonging to the genus *Halorubrum* accounted for 65.48% of all archaea specific counts. The relative abundance of all archaeal RSVs at family level is depicted in Figure 8B.

#### Alpha Diversity

For alpha diversity, the indices “Observed,” “InvSimpson” (both residuals normally distributed, Shapiro–Wilk test: “Observed” *p* = 0.3994, “InvSimpson” *p* = 0.5301) and “Shannon” (residuals not normally distributed, Shapiro–Wilk test: *p* = 0.04203) were calculated. An analysis of variance (ANOVA) was used for testing significant differences between sites for the indices “Observed” and “InvSimpson.” For the Shannon index, the Kruskal–Wallis test was used. None of the indices indicated significant differences between the sites. This was also true for a comparison between the zones medial moraine (combining AMH and AML) and glacier terminus (combining ANR, WIP, *t*-test for normally distributed residuals of “Observed” and “InvSimpson” indices, Kruskal–Wallis test for “Shannon” index).

On average, 79.07 bacterial RSVs were detected in CHs and compared to samples from the medial moraine (mean 64.63 ± 29.26; AMH: 66.75 ± 33.84, AML: 62.5 ± 24.23) higher numbers were observed at the glacier terminus (mean 98.33 ± 45.63; ANR: 130.67 ± 36.26, WIP: 66 ± 27.58).

For archaea, the same diversity indices as for bacteria were calculated and distribution of residuals was tested for normality with the Shapiro–Wilk test (“Observed” *p* = 0.007758, “InvSimpson” *p* = 0.02033, “Shannon” *p* = 0.07927). Due to the absence of archaea in two samples, statistical comparisons were not attempted between single sites but between all samples from the medial moraine and from the glacier terminus. Neither the Kruskal–Wallis tests for the indices “Observed” and “InvSimpson” nor a *t*-test for index “Shannon” indicated significant differences between the two sites. In comparison with the bacterial RSV counts, the number of observed archaeal RSVs was an order of magnitude lower (4.25 ± 3.44) and the distribution was in the same range for the medial moraine (4 ± 4.28) and samples from the glacier terminus (4.6 ± 1.62). Representative boxplots for bacterial and archaeal alpha diversity are depicted in Supplementary Figure 6.

#### Beta Diversity

**FIGURE 9:**
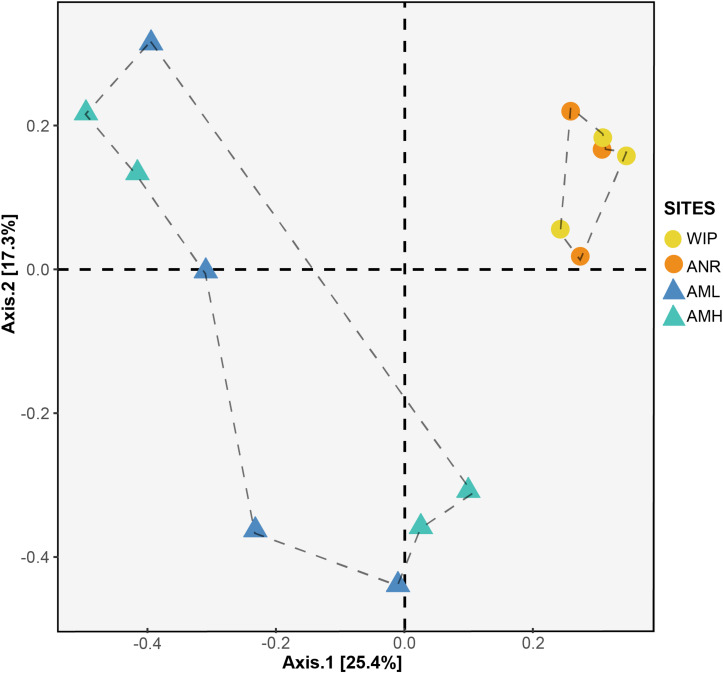
PCoA plot based on Bray–Curtis distance matrix calculated from the RSV table. Circles and triangles represent samples from the glacier terminus and the medial moraine, respectively. Dashed lines serve as a visual guidance.

To identify whether CH communities differed between sites, we performed an analysis of similarities (ANOSIM) based on a Bray–Curtis RSV dissimilarity matrix. CH communities between the zones medial moraine (combined AML and AMH) and glacier terminus (combined ANR and WIP) were significantly different (*p* = 0.04, 999 permutations) but dissimilarities between all four sites were not significant (*p* = 0.08, 999 permutations). This is corroborated by a Principal Coordinates Analysis (PCoA) depicted in Figure 9. Samples from the glacier terminus were more similar to each other than those along the medial moraine. CHs from the medial moraine formed two clusters that were not attributed to their spatial position at the glacier.

To identify key parameters that explain community variances, we performed permutational analysis of variance (PERMANOVA). The variance was mostly explained by depth (20.52%), mineral diversity (12.04%), total OM (10.47%) and the particle distribution in the size range 40–100 μm (7.31%) (Supplementary Table 2).

Further we performed a differential abundance analysis to identify RSVs that accounted for compositional differences between the medial moraine and the glacier terminus. These differences were attributed to 26 RSVs belonging to the phyla Actinobacteria (*n* = 3), Armatimonadetes (*n* = 1), Bacteroidetes (*n* = 7), Cyanobacteria (*n* = 2), Planctomycetes (*n* = 2), Proteobacteria (*n* = 10), and Saccharibacteria (*n* = 1). The complete taxonomy of these differentially abundant RSVs are shown in Supplementary Table 3.

ANOSIM for archaeal-related RSVs were neither significant for all four sites (*p* = 0.544, 999 permutations) nor for between the zones medial moraine and the glacier terminus (*p* = 0.537, 999 permutations). PERMANOVA and DESEQ2 were not applied to the archaeal dataset due to lower the amount of available data.

**FIGURE 10:**
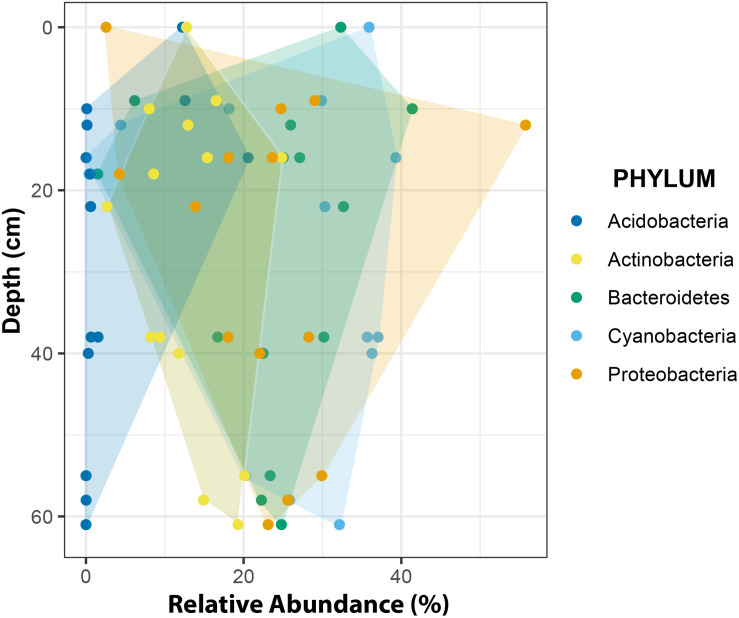
Relative abundance of major phyla across a depth gradient. The borders of the colored areas indicate the outermost data points.

At phylum level, changes in relative abundance along a depth gradient indicated that members of Acidobacteria and Actinobacteria occurred more frequently in shallow CHs while Cyanobacteria, Proteobacteria and Bacteroidetes trended toward more similar frequencies with increasing depths (Figure 10).

#### Core Microbiome of Cryoconite Holes

The core microbiome of CHs from the Anuchin Glacier was defined as RSVs with an average abundance across all sites ≥ 0.1%. For bacteria, the core microbiome consisted of 30 RSVs that were mainly affiliated with Cyanobacteria (23.30%), Bacteroidetes (9.07%), Actinobacteria (8.61%), Proteobacteria (7.69%), and others (combined 4.32%). The most abundant genera within the bacterial core microbiome were *Tychonema* (9.36%), *Chamaesiphon* (9.09%), RSVs affiliated with a not assigned genus belonging to Microbacteriaceae (6.46%), *Leptolyngbya* (4.86%), and *Polaromonas* (2.55%). From all 24 archaea-related RSVs only four were present across all sites with a relative abundance of ≥0.1%. These sequences belonged to the classes Methanomicrobia (39.88%) and Thermoplasmata (2.43%) and the most abundant RSVs could not be assigned at genus level.

Supplementary Figure 7 depicts all RSVs for bacteria and archaea exceeding the ≥0.1% threshold according to their occurrence from 1 (i.e., occurrence at only one site) to 4 sites (i.e., core microbiome).

## Discussion

### Mineral Composition Is Linked to Local Windblown Material

One aim of this study was to characterize the minerogenic composition of CHs across the Anuchin Glacier that is flanked by anorthite – norite alterations. We hypothesized that the CH minerogenic composition differed between the medial moraine and CH samples from other zones from the same glacier. Such dissimilarities may be considered as a proxy for differences in microbial community composition across the glacier. We found that particle size classes differed between sites and that minerals occurring at the glacier terminus (Albite, Anorthite, Biotite, Quartz, Magnesiohornblende) were also common in CHs along the medial moraine which were additionally enriched with other minerals (Sanidine, Grossular, Microcline, Sodium Aluminum Silicate, Rutile, Clinochlore, Chamosite, Laumontite). Some of these additional minerals were also identified approx. 15 km north of Lake Untersee at the Otradnaya Nunatak (Markl and Piazolo, 1998) which is considered as source material of the medial moraine. Similar geochemical compositions between the Nunatak and the medial moraine were also confirmed by Bormann and Fritzsche (1995). Therefore, CHs across the glacier can be classified according to a combination of their minerogenic contents and particle size distribution which is either linked to local and likely wind-blown material within the oasis (low mineral diversity) or to the further distant Otradnaya Nunatak (higher mineral diversity).

### Physical Characteristics of CHs Depends on Location

We established a temperature depth gradient time series to identify differences between sites with clear ice, dark-colored ice from the medial moraine and ice from the white ice patch. The temperature loggers were considered as a proxy for cryoconite *in situ* temperatures as the reflectivity of the loggers and the sediments were in a similar range (Supplementary Figure 1). As expected, dark-colored medial moraine ice was warmer than clear glacier ice and the highest average *in-situ* temperatures were recorded at the WIP site which is likely thermally influenced by Lake Untersee and hence may promote additional melting. Further, the rate of temperature declined with depth but varied between sites. This decline was higher in clear glacier ice than at the medial moraine and may be attributed to the absence of suppressing mineral material which enables the temperature wave unhindered penetration. The smallest differences over depth were found at WIP which therefore may not only enable deeper cryoconite layers but also less daily temperature fluctuations and hence more stable conditions. We found that CH proxy temperatures at least temporarily exceeded ambient air temperatures – especially between the surface and 0.5 depth. In comparison, CH temperatures at Canada Glacier were permanently higher than the local air temperature (Fountain et al., 2008; Bagshaw et al., 2011). However, the CH depths in these studies did not exceed 50 cm and a direct comparison with this study does not constitute meaningful information because different types of sensors were used and hence thermal inertia between the probes may not be comparable. However, even if considered as a relative measurement, differences between sites, depths and ambient temperature were evident and therefore provide valuable information for future temperature analyses.

Studies from the MDVs (Darcy et al., 2018) described CH size as “island size” implying that CHs are considered as islands across the supraglacial surface. Here they showed significant relations between size, depth and bacterial diversity. CHs were significantly shallower along the medial moraine (0 – 22 cm) compared to those at the glacier terminus (38 – 61 cm). Wharton et al. (1985) predicted that hole-deepening with respect to the ice surface slows down as the energy input required for melting decreases with depth until an equilibrium between surface ablation and hole-deepening rate is reached. Common equilibrium depths are reported between 30 and 50 cm (Gribbon, 1979). The hypothesis was supported by field experiments in the MDVs which further showed that CH equilibrium depths were reached by the end of the melt season (Fountain et al., 2008). The same study also concluded that the magnitude of subsurface melting depended on the optical properties of the overlaying ice. Such differences were also evident at the Anuchin Glacier. For example, the medial moraine contained micro-scale sized particles that visually darkened the ice and hence likely decreased the equilibrium depths by absorbing most solar irradiation before reaching the cryoconite layer. This would also explain the strongest de-coupling effect between *in-situ* proxy temperatures at the medial moraine and ambient air temperatures.

Further, the two sampling sites at the glacier terminus had distinctive depth characteristics. The deepest layers were found at the WIP site, possibly caused by heat transfer from Lake Untersee to the white ice matrix and its embedded CHs. Moreover, ablation rates north of Lake Untersee are lower than within the oasis (Scheinert et al., 2006) and hence may reduce the duration required to reach equilibrium depths. Additionally, satellite based observations showed that surface temperatures generally decrease with increasing distance from the oasis (Supplementary Figure 8), suggesting that equilibrium depths may also depend on the spatial location.

In comparison, mean CH depths from five glaciers in Southern Victoria Land covering an altitudinal gradient from 30 to 950 m above sea level were 33–41 cm deep and did not differ significantly between glaciers. However, only CHs with diameter > 30 cm (and hence deeper CH) were targeted in their study (Webster-Brown et al., 2015).

Fountain et al. (2004) argued that CH diameters also depended on the initial sediment load that initialized the CH formation. This was observed at several glaciers across the MDVs (Porazinska et al., 2004). We found that CH diameters were different between sites and that diameters increased with the mineral diversity (*p* = 0.056, Adj. *r*^2^ = 0.21) which was considered as an indicator whether the sediment load derived from vicinal windblown material or from the medial moraine and probably from adjacent steep slopes.

### Extremely Low Nutrient Levels Do Not Support High Rates of Microbial Activity in Antarctic Cryoconite Holes

Landscape position impacts nutrient stoichiometry in the MDVs soils (Barrett et al., 2007) and such spatial patterns were also observed at the Anuchin Glacier. Total carbon contents were very low and significantly different between the zones and single sites, respectively. Average contents per site ranged from 0.58 to 2.52 mg g^–1^ and were in the same order of magnitude as reported from Canada Glacier in MDVs (Telling et al., 2014) but slightly higher than those near Princess Elisabeth station (Lutz et al., 2019). Also total nitrogen contents differed between sites and average values from each site ranged from 0.07 to 0.23 mg g^–1^ which is comparable with CHs from the nearby Sør Rondane mountains in Dronning Maud Land (Lutz et al., 2019). However, total nitrogen contents were 2 – 3 fold higher at the glacier terminus than further up-glacier. Total phosphorus was generally low and did not differ between the sites. Webster-Brown et al. (2015) reports about phosphorous levels in MDV entombed CHs close to detection limits. At Darwin Glacier (Victoria Land) they resulted the same low nutrient concentrations (Webster-Brown et al., 2010), however, these values derive from meltwater biogeochemistry.

Bacterial carbon production in CHs from the Anuchin Glacier did not significantly differ between sites or zones and was exceptionally low (4.65^∗^10^–03^± 1.33^∗^10^–02^ ng C g^–1^ h^–1^). Thus far, these values represent the lowest BCP rates measured in Antarctic CHs. In comparison, other studies reported BCP rates ranging from 23.4 ng C g^–1^ h^–1^ at Canada, Commonwealth and Taylor Glaciers (Anesio et al., 2010) to 11.2 ng C g^–1^ h^–1^ in the Patriot Hills. BCP in CH water from the Vestfold Hills was 1.58 ng C g^–1^ h^–1^ (Foreman et al., 2007). The reason for these low rates is the subject of ongoing studies.

Cell abundances did not show any pattern across the glacier and the depth gradient. Average numbers per site were in the order of 10^7^ cells g dw^–1^. These numbers are comparable with those from a blue ice CH system (Hodson et al., 2013) and cryoconite from the Patriot Hills (Anesio et al., 2010). Abundances reported from coastal Antarctica (Grzesiak et al., 2015) and the Taylor Valley (Foreman et al., 2007) were one and two orders of magnitude lower, respectively. Higher counts at the Anuchin Glacier may be explained by the application of an efficient cell-dislodgement protocol (Duhamel and Jacquet, 2006). To our knowledge, this protocol has not been used in other Antarctic CH studies yet and hence, the comparison of cell numbers is trustworthy.

Total OM contents in CHs were significantly higher at the glacier terminus (=deeper CHs). This suggests that OM may accumulate over time in deeper CHs, assuming that CHs across the glacier reach similar equilibrium depths. For statistical analysis we identified and consequently removed one outlier (CRY-7) that had a 5.8 times higher OM content as the overall observed mean value and exceeded the interquartile range for this parameter. We partly attribute this exceptional high mass loss to the presence of hydrogen-bonded (opposed to Ca-bonded) water in Laumontite (Ca[Al_2_Si_4_O_12_] × 4–4.5 H_2_0) which was only detected in this sample. When heated, the mineral gradually releases structural water from 100 to 750°C (Földvári, 2011). As a consequence, loss on ignition (LOI) measurements in low biomass samples should be accompanied by mineral analyses to correctly interpret suspicious data.

Hodson et al. (2013) did not find significant correlations between BCP and OM contents in CHs and despite the low BCP rates at the Anuchin Glacier, OM fractions were in a comparable range with other studies. For example, CHs collected in the vicinity of Princess Elisabeth station had mean and maximum mass losses of 0.64 and 1.12% when heated up to 1000°C, respectively (Lutz et al., 2019). We re-calculated our TGA results (38 – 1000°C) for a direct comparison. The overall mean loss on ignition without outlier sample CRY-7 was in a similar range (mean: 0.48%, max: 1.07%). Therefore, CHs at the next closest study site (∼353 km west of Lake Untersee Oasis) are comparable with respect to their mass-loss properties. OM contents in CHs from a blue ice zone were slightly higher and ranged from 0.78 to 1.99% (Hodson et al., 2013).

By considering the higher cell number which is possibly due to the more efficient dislocation protocol and the low productivity we assume that the microbial activity of the respective CH communities must be extremely low. Moreover, the exceptional low content of OM would not provide the required sources therefore as well as the extremely restricted availability of liquid water.

### Bacterial Phyla – “The Usual Suspects” Found in Antarctic Cryoconite Holes

In total, 92% of all RSV counts belonged to Proteobacteria, Cyanobacteria, Bacteroidetes, Actinobacteria, and Acidobacteria which are mirrored elsewhere in Antarctic environments (e.g., Christner et al., 2003; Webster-Brown et al., 2015). However, phyla with the most ribosomal sequence variants were Proteobacteria, Bacteroidetes, Actinobacteria, Planctomycetes, and Cyanobacteria. The presence of multiple sequence variants of the same genera may indicate a certain molecular plasticity in context with environmental conditions (Tikhonov et al., 2015).

Proteobacteria had the highest mean relative abundance in all samples (26.54%) and were mainly composed of Betaproteobacteria, Gammaproteobacteria, and Alphaproteobacteria. The most abundant RSV within this phylum was *Silanimonas* sp. which was also found in CHs of the Taylor Glacier in the MDVs (Christner et al., 2003; GenBank: AY124351.1: 99.62% similarity). The second most abundant RSV was the Alphaproteobacterium *Sphingomonas* sp. which was found in hypolithic microbial communities of quartz rocks from Miers Valley (Khan et al., 2011; GenBank: HQ197614.1, 99.63% similarity) and the ice cover of Lake Vida (Mosier et al., 2007; GenBank: DQ521492.1, 99.63% similarity). Another abundant RSV was *Polaromonas* sp. (Betaproteobacteria) that is also known from other polar regions and was identified in sediments of Lake Bonney (Tang et al., 2013, GenBank: JX948739.1, 99.26% similarity).

Cyanobacteria frequently occur in extreme environments and are known as important key members in supraglacial and other cold terrestrial zones (Vincent, 2000; Namsaraev et al., 2010; Chrismas et al., 2015). CHs from the Anuchin Glacier were also dominated by them (25,45%). The most abundant cyanobacterial RSVs were also found in soils of the Wright Valley (*Tychonema*: GenBank: KM052830.1; 100% similarity), Antarctic snow (Lopatina et al., 2013; GenBank: JX855325., 99.63% similarity). Filamentous Cyanobacteria play a key role in cryoconite granule formation (Jungblut and Vincent, 2017) and the granule size shapes the microbial community composition (Uetake et al., 2019). Here, typical cryoconite granule engineers (*Phormidium*, *Chamaesiphon*) were present but the granules were neither observed in other Antarctic regions. This might be due to a substantial lack of continuous flow of melt water along supraglacial surfaces which cause the original particles to roll in the flow where cells are attached on the outer side.

The main classes of Bacteroidetes in CHs were Cytophagia (dominated by *Algoriphagus* sp.) and Sphingobacteria (dominated *Ferruginibacter*). Similar *Algoriphagus* sequences were found in soils of the Antarctic Darwin Mountains (Aislabie et al., 2013; GenBank: KC442505.1, similarity: 98.90%) and *Ferruginibacter* is also known from other glacio-lacustrine Antarctic settings (GenBank: KP012224.1, similarity 99.63%).

Actinobacteria and Acidobacteria made up 16.58% of all counts. The most frequent sequences belonged to *Chryseoglobus* sp. and *Granulicella* sp., respectively. Similar *Chryseoglobus* signatures were found about 353 km west of Lake Untersee Oasis, nearby Utsteinen mountain (GenBank: FR682678.1; similarity: 98.90%). *Granulicella*-like sequences were reported from soils at South Georgia (Yergeau et al., 2007; GenBank: EF221026.1, similarity: 100%).

The overall phyla distribution changed with depth. Compared to shallow CHs the variability of relative abundances decreased in deeper CHs. This observation may be explained in two ways. First, environmental conditions in deeper CHs are more stable. For example, the temperature fluctuations are smaller than at the surface (e.g., Figure 4) because more light is attenuated by the overlaying ice cover (Bagshaw et al., 2016). This implies that approaching the CH equilibrium depth also reduces biotic inoculation by melting of surrounding ice and hence shapes more stable microbial communities. Secondly, CH depths are often linked with the isolation age, i.e., being disconnected from the atmosphere by the formation on an ice lid (Fountain et al., 2004; Bagshaw et al., 2007; Lutz et al., 2019). It is also likely that this isolation age may play a role in the formation of stable microbial communities in CHs.

Compositional differences in archaeal communities are linked with the selection of suitable primers and subsequent amplification steps (Pausan et al., 2019). In this study, the universal primer approach resulted in 1,588 archaea- related counts and identified Archaea in 57% of the samples. These numbers were significantly improved by the use of archaea-targeting primers 344f and 915r (Klindworth et al., 2013) and subsequent nested PCR approach with the primer S-D-Arch-0519-a-S-15 (Koskinen et al., 2017). Overall, archaea were detected in 85% of the samples. Also, the number of detected RSVs increased from 18 to 24 ribosomal sequence variants.

All archaea-specific sequences in CHs belonged to the classes Halobacteria, Methanomicrobia and Thermoplasmata within the phylum Euryarchaeota. By far the most abundant genus was *Halorubrum* (Halobacteria) that was affiliated to nine ribosomal sequence variants but more than 97% of all counts were attributed to one RSV. The most similar blasted sequence derived from the hypersaline Deep Lake (Mou et al., 2012; GenBank:NR_117806.1, similarity: 89,14%). Blast search did not result in more similar sequences for the second and third-most abundant sequences.

To our surprise we found only two other Antarctic CH studies that reported Archaea in their samples. Cameron et al. (2012) compared CHs from polar regions and showed that archaea were only detectable in Antarctic CHs and that most reads were affiliated with Thaumarchaeota and the classes Methanobacteriaceae and Methanomicrobia within the phylum Euryarchaeota. Further, about 4% of all CH samples nearby the East-Antarctic Princess Elisabeth station harbored archaea and were classified as *Nitrososphaera* (Thaumarchaeota) (Lutz et al., 2019). Hence, this study provided the first evidence of the classes Halobacteria and Thermoplasmata in Antarctic CHs. This low detection rate might also be a result of the missing approach with nested PCR which improves the identification as shown in this study.

### The Core Microbiome of Cryoconite Holes – A Matter of Definition

In this study, RSVs that were considered as part of the core microbiome had to fulfill two requirements: The mean relative abundance of all samples had to be ≥0.1% and the sequences had to occur at all sites. The first criterium was met by 67 sequences and after applying the second criterium only 30 RSVs remained in the dataset. Compared to the overall dominance of Proteobacteria in terms of relative abundance and RSV numbers, the core microbiome was dominated by Cyanobacteria, followed by Bacteroidetes, Actinobacteria, and Proteobacteria.

Sequence blasting showed that most RSVs of the CH core microbiome were similar to sequences that are already known from Antarctic environments. For example, cyanobacterial RSVs that were assigned to the genus *Chamaesiphon* were highly similar to those found in microbial mats of Lake Hoare (Hawes et al., 2016; GenBank: KU230337.1, 99.63% similarity), in CHs from the Lower Wright Glacier (Webster-Brown et al., 2015; GenBank: KT424939.1 99.26% similarity) and in dark soils of a glacier forefield at the Larsemann Hills (Bajerski and Wagner, 2013; GenBank: JX172501.1, 99.26% similarity). Further, RSVs affiliated with the cyanobacterial genus *Tychonema* were also found at the nearby Sør Rondane Mountains (GenBank: HM101190.1, 100% similarity) and in microbial mats of Lake Fryxell (GenBank: AY151751.1, similarity: 100%). Also RSVs assigned to the genus *Leptolyngbya* were highly similar to those retrieved from CH of the Diamond Glacier in Southern Victoria Land (Webster-Brown et al., 2015; GenBank: KT424929.1, similarity: 99.26%).

RSVs that belonged to Actinobacteria (genera Chryseoglobus, Cryobacterium, and Marisediminicola) were also found in the ice cover of Lake Vida (Mosier et al., 2007; GenBank: DQ521497.1, similarity: 99.63%), in soils at Robert Island (GenBank: MT072015.1, similarity: 99.63%) and in sediments of Lake Bonney (Tang et al., 2013; GenBank: JX948474.1, similarity: 99.63%), respectively.

The CH core microbiome was also composed of the genera *Thermomonas* (Gammaproteobacteria), *Polaromonas* (Betaproteobacteria) and *Rubellimicrobium* (Alphaproteobacteria) that were similar to those found in shallow groundwaters at Cape Hallett (Aislabie et al., 2009; GenBank: FJ164057.1, similarity: 97.07%), surface waters of Lake Limnopolar at Byers Peninsula (Papale et al., 2017; GenBank: KF928875.1, similarity: 99.63%) and sediments of Lake Bonney (Tang et al., 2013; GenBank: JX948518.1, similarity: 98.16%), respectively. Further, core RSVs assigned to the genus *Hymenobacter* (Bacteroidetes) were found in sediments of the Onyx River within the Wright Valley (Zeglin et al., 2011; GenBank: EU869634.1, similarity: 97.06%). These observations indicate that members of the CH core microbiome of the Anuchin Glacier also prevail in other habitats spread across Antarctica and that they cope with a wide range of environmental conditions.

Despite the lack of Archaea in 2 CHs, four RSVs passed our core microbiome criteria. These sequences belonged to unknown genera of the families Methanomicrobiales, Thermoplasmatales and to Methanocellales (*Rice_Cluster_I*). Blast results revealed only distant relatives within Antarctica which were present in Lake Fryxell (Karr et al., 2006; GenBank: AY299382.1, similarity: 95.09%), in endolithic communities from the Miers Valley (GenBank: KC476273.1, similarity: 84.50%) and in marine hydrothermal sediments (GenBank: FM868021.1, similarity: 87.4%).

A direct comparison of CH core microbiomes is hampered for two reasons. First, data availability of Antarctic CHs core microbiomes is scarce. Second, differences in methodological approaches limit such comparisons. For example, compared to the use of operational taxonomic units (OTUs, similarity threshold 97%; e.g., Lutz et al., 2019), the use of RSVs (e.g., this study) leads to an increased number of total sequences because RSVs are more exact than OTUs (Callahan et al., 2016). Consequently, read counts are allocated to more amplicon sequence variants and therefore the probability for a sequence being detected at all sites is reduced. As an example, the CH core microbiomes of the Anuchin Glacier and from a site in the Sør Rondane mountains (Lutz et al., 2019) broadly match at phylum level but differences at higher taxonomic resolution are evident. For instance, Lutz et al. (2019) found members of the family Sphingomonadaceae (Alphaproteobacteria) in their core microbiome but in the present study none of the 17 Sphingomonadaceae assigned RSVs passed our core microbiome criteria. This example highlights that the core microbiome is a matter of definition (e.g., prevalence levels, site vs. sample occurrence) but it also depends on underlying methodological approaches (e.g., primer selection, bioinformatic pipelines).

### Inoculation Sources and Their Role for Cryoconite Hole Establishment

Samples from the glacier terminus shared characteristics for most biogeochemical features and therefore clustered together in the PCA (Figure 7). The cluster formation was mainly attributed to the variables depth, diameter, total nitrogen, and total carbon. CHs from the medial moraine had a higher variability and cluster formation was not evident. Independent of this metadata analysis a principal coordinates analysis (Figure 9) revealed a striking similar pattern: Microbial communities from the glacier terminus were similar to each other while samples within the medial moraine showed greater differences between single samples.

Differential abundance analysis (DESEQ2) showed that the key differences in microbial community composition between the medial moraine and the glacier terminus samples were mainly caused by 26 RSVs belonging to the phyla Actinobacteria (*n* = 3), Armatimonadetes (*n* = 1), Bacteroidetes (*n* = 7), Cyanobacteria (*n* = 2), Planctomycetes (*n* = 2), Proteobacteria (*n* = 10), and Saccharibacteria (*n* = 1). Some of those sequences were also found elsewhere in Antarctica. For example, *Spirosoma* sp. was found along in ice-free soils near the Belgium Princess Elisabeth station which is only ∼350 km away from the study site (GenBank: MN031263.1, MN031264.1).

CHs form independently from each other and therefore may resemble discrete ecosystems. However, CH communities from the MDVs were similar to those from the surrounding soils (Christner et al., 2003). Contradictory, another study from the same region concluded that the CH composition did not mirror biota that was found in the surrounding (Porazinska et al., 2004). Also, CHs in close proximity tend to be more similar compared to further distant ones (Darcy et al., 2018) and hence clustered according to their location, suggesting local inoculum sources and environmental conditions (Lutz et al., 2019) which is also valid for other extreme Antarctic environments such as supraglacial ponds or soils (Webster-Brown et al., 2010; Lee et al., 2012).

Potential microbial source inocula at the Anuchin Glacier are numerous and varied, including those originating from surrounding ice and soils. Also, differences in mineral diversity between the medial moraine and the glacier terminus may be considered as an indicator for separate local inoculum sources. Further, re-surfacing wind-blown microbial mats from Lake Untersee may re-colonize CHs at the Anuchin glacier and consequently return to the lake by melt processes (Weisleitner et al., 2019b). Further, similarities between genera that were found at “nearby” Sør Rondane mountains (Obbels et al., 2016; Lutz et al., 2019) and more distant Antarctic sites (e.g., Aislabie et al., 2013; Lopatina et al., 2013) indicate long-range aeolian biotic sources that reach Lake Untersee Oasis.

The dynamics of bacterial and archaeal communities presented in this study are most likely controlled by a combination of environmental factors. One of these drivers for microbial evolution can be identified by viruses which play a key role in the microbial loop (Anesio and Bellas, 2011). As reported for Arctic CHs, they increase bacterial mortality by lysis and therefore promote nutrient re-utilization (Bellas et al., 2013). Also, transplantation experiments have shown that viruses from CHs also infect microbial communities of an adjacent proglacial lake (Anesio et al., 2007). This suggests that the impact of a potential CH viriome is not limited to the supraglacial zone of the Anuchin Glacier but likely controls microbial communities within adjacent Lake Untersee. However, despite the evidence of phage infections within the water column of Lake Untersee (Filippova et al., 2013), neither the genetic viral diversity nor their sources and interactions are presently known.

## Conclusion

CHs at the Anuchin Glacier are considered as important biotic inoculum sources for adjacent Lake Untersee ecosystem (Weisleitner et al., 2019b). Here, we further identified differences in abiotic and biotic characteristics between CHs occurring along the medial moraine and the glacier terminus and defined the bacterial and archaeal core microbiome from this habitat.

Based on the mineral diversity distribution across sites, we accept the initial hypothesis that minerogenic composition differed between the medial moraine and the glacier terminus. These differences also indicate distinctive biotic inoculum sources (e.g., biota attached to windblown dust from within the oasis vs. sediments from Otradnaja Nunatak outside the oasis).

Our observations partially support the second hypothesis that community composition, OM contents and BCP rates are mirrored by the depth of the cryoconite layer. Depth was the most important variable that explained differences in the community composition. Also, both OM fractions (labile and stable) were significantly different between shallow CHs along the medial moraine and deeper ones that occurred at the glacier terminus. However, BCP insignificantly trended toward higher rates in shallow CHs and hence did not fully underpin our initial hypothesis. Further, the reported BCP rates represent the lowest records known from Antarctic CHs.

We used temperature loggers as proxies for *in situ* cryoconite temperatures. Despite similar reflectivity of cryoconite and the loggers, thermal inertia of the loggers likely underestimated the maximum temperatures within the ice. However, a relative comparison between the sites is valid. We demonstrated that temperature dynamics differed between dark colored ice that was interspersed with particulates, ice that appeared macroscopically free of particles and glacial ice that was embedded in the ice cover of Lake Untersee, respectively. Further, differences in CH depth distribution could be explained by a combination of optical properties of the overlaying ice and the temperature profiles which lead to distinct environmental conditions across the glacier.

We identified bacteria that are commonly found in CHs. However, this is the first study that identified members of the archaeal classes Halobacteria and Thermoplasmata in Antarctic CHs and to our knowledge the percentage of CHs that harbored archaea was higher than reported elsewhere. The role of archaea in CHs at the Anuchin Glacier are poorly studied and hence should be subject of future studies in context with Lake Untersee Oasis.

## Data Availability Statement

Sequence data presented in this manuscript is deposited in the NCBI database under the accession number SRP145579 and was published among other data in Weisleitner et al. (2019b). Accession numbers linking to single samples and their according primer sets are found in Supplementary Table 1 of this manuscript.

## Author Contributions

KW designed the study, collected all the samples, and performed all the *in situ* measurements during an expedition that was led by DA. SU performed the TGA and XRD measurements and acquired the scanning electron microscopy images. KW and AP did the microbial community analysis. KW did the statistical analysis of microbial data and metadata. KW and BS wrote most of the manuscript (KW: main contributor) that was reviewed by all authors.

## Conflict of Interest

The authors declare that the research was conducted in the absence of any commercial or financial relationships that could be construed as a potential conflict of interest.

## References

[B1] Aislabie J. M. Lau A. Dsouza M. Shepherd C. Rhodes P. Turner S. J. (2013). Bacterial composition of soils of the Lake Wellman area, Darwin Mountains, Antarctica. *Extremophiles* 17 775–786. 10.1007/s00792-013-0560-6 23820800

[B2] Aislabie J. Ryburn J. Sarmah A. (2009). Culturable microbes in shallow groundwater underlying ornithogenic soil of Cape Hallett, Antarctica. *Can. J. Microbiol*. 55, 12–20. 10.1139/W08-118 19190697

[B3] Andersen D. T. McKay C. P. Lagun V. (2015). Climate conditions at perennially ice-covered Lake Untersee, East Antarctica. *J. Appl. Meteorol. Climatol.* 54 1393–1412. 10.1175/jamc-d-14-0251.1

[B4] Andersen D. T. Sumner D. Y. Hawes I. Webster-Brown J. Mckay C. P. (2011). Discovery of large conical stromatolites in Lake Untersee, Antarctica. *Geobiology* 9 280–293. 10.1111/j.1472-4669.2011.00279.x 21504538

[B5] Anesio A. M. Bellas C. M. (2011). Are low temperature habitats hot spots of microbial evolution driven by viruses? *Trends Microbiol.* 19 52–57. 10.1016/j.tim.2010.11.002 21130655

[B6] Anesio A. M. Hodson A. J. Fritz A. Psenner R. Sattler B. (2009). High microbial activity on glaciers: importance to the global carbon cycle. *Glob. Change Biol.* 15 955–960. 10.1111/j.1365-2486.2008.01758.x

[B7] Anesio A. M. Mindl B. Laybourn-Parry J. Hodson A. J. Sattler B. (2007). Viral dynamics in cryoconite holes on a high Arctic glacier (Svalbard). *J. Geophys. Res.* 112:G04S31 10.1029/2006JG000350

[B8] Anesio A. M. Sattler B. Foreman C. Telling J. Hodson A. Tranter M. (2010). Carbon fluxes through bacterial communities on glacier surfaces. *Ann. Glaciol.* 51 32–40. 10.3189/172756411795932092

[B9] Bagshaw E. A. Tranter M. Fountain A. G. Welch K. A. Basagic H. Lyons W. B. (2007). Biogeochemical evolution of cryoconite holes on Canada Glacier, Taylor Valley, Antarctica. *J. Geophys. Res.* 112:G04S35 10.1029/2007JG000442

[B10] Bagshaw E. A. Tranter M. Fountain A. G. Welch K. Basagic H. J. Lyons W. B. (2013). Do cryoconite holes have the potential to be significant sources of C, N, and P to downstream depauperate ecosystems of Taylor Valley, Antarctica? *Arct. Antarct. Alp. Res*. 45, 440–454. 10.1657/1938-4246-45.4.440

[B11] Bagshaw E. A. Tranter M. Wadham J. L. Fountain A. G. Dubnick A. Fitzsimons S. (2016). Processes controlling carbon cycling in Antarctic glacier surface ecosystems. *Geochem. Persp. Lett.* 2 44–54. 10.7185/geochemlet.1605 15074972

[B12] Bagshaw E. A. Tranter M. Wadham J. L. Fountain A. G. Mowlem M. (2011). High-resolution monitoring reveals dissolved oxygen dynamics in an Antarctic cryoconite hole. *Hydrol. Process.* 25 2868–2877.

[B13] Bajerski F. Wagner D. (2013). Bacterial succession in Antarctic soils of two glacier forefields on Larsemann Hills, East Antarctica. *FEMS Microbiol. Ecol*. 85, 128–142. 10.1111/1574-6941.12105 23480659

[B14] Barrett J. E. Virginia R. A. Lyons W. B. McKnight D. M. Priscu J. C. Doran P. T. (2007). Biogeochemical stoichiometry of Antarctic Dry Valley ecosystems. *J. Geophys. Res.* 112:G01010 10.1029/2005JG000141

[B15] Bellas C. Anesio A. Telling J. Stibal M. Tranter M. Davis S. (2013). Viral impacts on bacterial communities in Arctic cryoconite. *Environ. Res. Lett.* 8:045021.

[B16] Bormann P. Fritzsche D. (1995). *The Schirmacher Oasis, Queen Maud Land, East Antarctica, and Its Surroundings.* Gotha: Peterm. Geogr. Mitt. Erg, 448.

[B17] Brandmaier A. M. (2015). pdc: an R package for complexity-based clustering of time series. *J. Stat. Softw.* 67 1–23. 10.18637/jss.v067.i05

[B18] Callahan B. J. McMurdie P. J. Rosen M. J. Han A. W. Johnson A. J. A. Holmes S. P. (2016). DADA2: High-resolution sample inference from Illumina amplicon data. *Nat. Methods* 13 581–583. 10.1038/nmeth.3869 27214047 PMC4927377

[B19] Cameron K. A. Hodson A. J. Osborn A. M. (2012). Structure and diversity of bacterial, eukaryotic and archaeal communities in glacial cryoconite holes from the Arctic and the Antarctic. *FEMS Microbiol. Ecol.* 82 254–267. 10.1111/j.1574-6941.2011.01277.x 22168226

[B20] Caporaso J. G. Lauber C. L. Walters W. A. Berg-Lyons D. Huntley J. Fierer N. (2012). Ultra-high-throughput microbial community analysis on the Illumina HiSeq and MiSeq platforms. *ISME J.* 6 1621–1624. 10.1038/ismej.2012.8 22402401 PMC3400413

[B21] Chrismas N. A. M. Anesio A. M. Sánchez-Baracaldo P. (2015). Multiple adaptations to polar and alpine environments within cyanobacteria: a phylogenomic and Bayesian approach. *Front. Microbiol.* 6:1070. 10.3389/fmicb.2015.01070 26528250 PMC4602134

[B22] Christner B. C. Kvitko B. H. Reeve J. N. (2003). Molecular identification of bacteria and eukarya inhabiting an Antarctic cryoconite hole. *Extremophiles* 7 177–183. 10.1007/s00792-002-0309-0 12768448

[B23] Cook J. Edwards A. Takeuchi N. Irvine-Fynn T. (2016). Cryoconite: the dark biological secret of cryosphere. *Prog. Phys. Geogr.* 40 66–111. 10.1177/0309133315616574

[B24] Darcy J. L. Gendron E. Sommers P. Porazinska D. L. Schmidt S. K. (2018). Island biogeography of cryoconite hole bacteria in Antarctica’s Taylor Valley and around the world. *Front. Ecol. Evol.* 6:180 10.3389/fevo.2018.00180

[B25] Duhamel S. Jacquet S. (2006). Flow cytometric analysis of bacteria-and virus-like particles in lake sediments. *J. Microbiol. Methods* 64 316–332. 10.1016/j.mimet.2005.05.008 16081175

[B26] Edwards A. Pachebat J. A. Swain M. Hegarty M. Hodson A. J. Irvine-Fynn T. D. (2013). A metagenomic snapshot of taxonomic and functional diversity in an alpine glacier cryoconite ecosystem. *Environ. Res. Lett.* 8:035003 10.1088/1748-9326/8/3/035003

[B27] Faucher B. Lacelle D. Fisher D. Andersen D. McKay C. (2019). Energy and water mass balance of Lake Untersee and its perennial ice cover, East Antarctica. *Antarct. Sci.* 31 271–285. 10.1017/S0954102019000270

[B28] Filippova S. N. Surgucheva N. A. Kulikov E. E. Sorokin V. V. Akimov V. N. Bej A. K. (2013). Detection of phage infection in the bacterial population of Lake Untersee (Antarctica). *Microbiology* 82 383–386. 10.1134/S0026261713030041 24466739

[B29] Földvári M. (2011). *Handbook of Thermogravimetric System of Minerals and its Use in Geological Practice*, Vol. 213 Budapest: Geological Institute of Hungary, 1–180.

[B30] Foreman C. M. Sattler B. Mikucki J. A. Porazinska D. L. Priscu J. C. (2007). Metabolic activity and diversity of cryoconites in the Taylor Valley, Antarctica. *Aquat. Geochem.* 11 391–412. 10.1007/s10498-004-7373-2

[B31] Fortner S. K. Tranter M. Fountain A. Lyons W. B. Welch K. A. (2005). The geochemistry of supraglacial streams of Canada Glacier, Taylor Valley (Antarctica), and their evolution into proglacial waters. *Aquat. Geochem.* 11 391–412.

[B32] Fountain A. G. Nylen T. H. Tranter M. Bagshaw E. (2008). Temporal variations in physical and chemical features of cryoconite holes on Canada Glacier, McMurdo Dry Valleys, Antarctica. *J. Geophys. Res. Biogeosci.* 113:G01S92.

[B33] Fountain A. G. Tranter M. Nylen T. H. Lewis K. J. Mueller D. R. (2004). Evolution of cryoconite holes and their contribution to meltwater runoff from glaciers in the McMurdo Dry Valleys, Antarctica. *J. Glaciol.* 50 35–45.

[B34] Gribbon P. (1979). Cryoconite Holes on Sermikavsak, West Greenland. *J. Glaciol.* 22 177–181. 10.3189/S0022143000014167

[B35] Grzesiak J. Zdanowski M. K. Górniak D. Świątecki A. Aleksandrzak-Piekarczyk T. Szatraj K. (2015). Microbial community changes along the ecology glacier ablation zone (King George Island, Antarctica). *Polar Biol.* 38 2069–2083.

[B36] Hamilton N. E. Ferry M. (2018). ggtern: ternary diagrams using ggplot2. *J. Stat. Softw.* 87 1–17.

[B37] Hawes I. Jungblut A. D. Obryk M. K. Doran P. T. (2016). Growth dynamics of a laminated microbial mat in response to variable irradiance in an Antarctic lake. *Freshw. Biol*. 61, 396–410. 10.1111/fwb.12715

[B38] Hermichen W. D. Kowski P. Wand U. (1985). Lake Untersee, a first isotope study of the largest freshwater lake in the interior of East Antarctica. *Nature* 315 131–133. 10.1038/315131a0

[B39] Hodson A. Anesio A. M. Tranter M. Fountain A. Osborn M. Priscu J. (2008). Glacial ecosystems. *Ecol. Monogr.* 78 41–67. 10.1890/07-0187.1

[B40] Hodson A. Paterson H. Westwood K. Cameron K. Laybourn-Parry J. (2013). A blue-ice ecosystem on the margins of the East Antarctic ice sheet. *J. Glaciol.* 59 255–268. 10.3189/2013JoG12J052

[B41] Howat I. Morin P. Porter C. Noh M. J. (2018). *The Reference Elevation Model of Antarctica.* Washington, DC: Harvard Dataverse.

[B42] Jungblut A. D. Vincent W. F. (2017). “Cyanobacteria in Polar and Alpine Ecosystems,” in *Psychrophiles: From Biodiversity to Biotechnology*, ed. Margesin R. (Cham: Springer), 181–206.

[B43] Karr E. A. Ng J. M. Belchik S. M. Sattley W. M. Madigan M. T. Achenbach L. A. (2006). Biodiversity of methanogenic and other Archaea in the permanently frozen Lake Fryxell, Antarctica. *Appl. Environ. Microbiol.* 72 1663–1666. 16461723 10.1128/AEM.72.2.1663-1666.2006 PMC1392947

[B44] Khan N. Tuffin M. Stafford W. Cary C. Lacap D. C. Pointing S. B. (2011). Hypolithic microbial communities of quartz rocks from Miers Valley, McMurdo Dry Valleys, Antarctica. *Polar Biol.* 34 1657–1668.

[B45] Kirchman D. (2001). Measuring bacterial biomass production and growth rates from leucine incorporation in natural aquatic environments. *Methods Microbiol.* 30 227–237.

[B46] Klindworth A. Pruesse E. Schweer T. Peplies J. Quast C. Horn M. (2013). Evaluation of general 16S ribosomal RNA gene PCR primers for classical and next-generation sequencing-based diversity studies. *Nucleic Acids Res.* 41:e1. 10.1093/nar/gks808 22933715 PMC3592464

[B47] Klymiuk I. Bambach I. Patra V. Trajanoski S. Wolf P. (2016). 16S based microbiome analysis from healthy subjects’ skin swabs stored for different storage periods reveal phylum to genus level changes. *Front. Microbiol.* 7:2012. 10.3389/fmicb.2016.02012 28066342 PMC5167739

[B48] Koskinen K. Pausan M. R. Perras A. K. Beck M. Bang C. Mora M. (2017). First insights into the diverse human archaeome: specific detection of Archaea in the gastrointestinal tract, lung, and nose and on skin. *mBio* 8:e00824-17. 10.1128/mBio.00824-17 29138298 PMC5686531

[B49] Lee C. K. Barbier B. A. Bottos E. M. McDonald I. R. Cary S. C. (2012). The Inter-Valley Soil Comparative Survey: the ecology of Dry Valley edaphic microbial communities. *ISME J.* 6 1046–1057. 10.1038/ismej.2011.170 22170424 PMC3329106

[B50] Leslie A. (2011). *The Arctic Voyages of Adolf Erik Nordenskiöld.* Cambridge: Cambridge University Press, 1858–1879. 10.1017/CBO9781139151597

[B51] Lopatina A. Krylenkov V. Severinov K. (2013). Activity and bacterial diversity of snow around Russian Antarctic stations. *Res. Microbiol.* 164 949–958. 10.1016/j.resmic.2013.08.005 24012540

[B52] Love M. I. Huber W. Anders S. (2014). Moderated estimation of fold change and dispersion for RNA-seq data with DESeq2. *Genome Biol.* 15:550. 10.1186/s13059-014-0550-8 25516281 PMC4302049

[B53] Lutz S. Ziolkowski L. A. Benning L. G. (2019). The biodiversity and geochemistry of Cryoconite Holes in Queen Maud Land, East Antarctica. *Microorganisms* 7:160. 10.3390/microorganisms7060160 31159414 PMC6616603

[B54] MacDonell S. Sharp M. Fitzsimons S. (2016). Cryoconite hole connectivity on the Wright Lower Glacier, McMurdo Dry Valleys, Antarctica. *J. Glaciol.* 62 714–724. 10.1017/jog.2016.62

[B55] Markl G. Piazolo S. (1998). Halogen-bearing minerals in syenites and high-grade marbles of Dronning Maud Land, Antarctica: monitors of fluid compositional changes during late-magmatic fluid-rock interaction processes. *Contrib. Mineral. Petrol.* 132 246–268.

[B56] Mauri M. Elli T. Caviglia G. Uboldi G. Azzi M. (2017). “RAWGraphs: a visualisation platform to create open outputs,” in *Proceedings of the 12th Biannual Conference on Italian SIGCHI Chapter*, (New York, NY: ACM), 28.

[B57] McMurdie P. J. Holmes S. (2013). Phyloseq: an R package for reproducible interactive analysis and graphics of microbiome census data. *PLoS ONE* 8:e61217. 10.1371/journal.pone.0061217 23630581 PMC3632530

[B58] McMurdie P. J. Holmes S. (2014). Waste not, want not: why rarefying microbiome data is inadmissible. *PLoS Comput. Biol*. 10:e1003531. 10.1371/journal.pcbi.1003531 24699258 PMC3974642

[B59] Morgan-Kiss R. M. Priscu J. C. Pocock T. Gudynaite-Savitch L. Huner N. P. (2006). Adaptation and acclimation of photosynthetic microorganisms to permanently cold environments. *Microbiol. Mol. Biol. Rev.* 70 222–252. 16524924 10.1128/MMBR.70.1.222-252.2006 PMC1393254

[B60] Mosier A. C. Murray A. E. Fritsen C. H. (2007). Microbiota within the perennial ice cover of Lake Vida, Antarctica. *FEMS Microbiol. Ecol.* 59 274–288. 17092309 10.1111/j.1574-6941.2006.00220.x

[B61] Mou Y. Z. Qiu X. X. Zhao M. L. Cui H. L. Oh D. Dyall-Smith M. L. (2012). *Halohasta litorea* gen. nov. sp. nov., and *Halohasta litchfieldiae* sp. nov., isolated from the Daliang aquaculture farm, China and from Deep Lake, Antarctica, respectively. *Extremophiles* 16 895–901. 10.1007/s00792-012-0485-5 23052830

[B62] Mueller D. R. Pollard W. H. (2004). Gradient analysis of cryoconite ecosystems from two polar glaciers. *Polar Biol.* 27 66–74.

[B63] Mueller D. R. Vincent W. F. Pollard W. H. Fritsen C. H. (2001). Glacial cryoconite ecosystems: a bipolar comparison of algal communities and habitats. *Nova Hedwigia Beiheft* 123 173–198.

[B64] Namsaraev Z. Mano M. Fernandez R. Wilmotte A. (2010). Biogeography of terrestrial cyanobacteria from Antarctic ice-free areas. *Ann. Glaciol.* 51 171–177. 10.3189/172756411795931930

[B65] Obbels D. Verleyen E. Mano M. J. Namsaraev Z. Sweetlove M. Tytgat B. (2016). Bacterial and eukaryotic biodiversity patterns in terrestrial and aquatic habitats in the Sør Rondane Mountains, Dronning Maud Land, East Antarctica. *FEMS Microbiol. Ecol.* 92:fiw041. 10.1093/femsec/fiw041 26936447

[B66] Oksanen J. Kindt R. Legendre P. O’Hara B. Stevens M. H. H. Oksanen M. J. (2007). The vegan package. *Community Ecol. Package* 10 631–637.

[B67] Papale M. Rizzo C. Villescusa J. A. Rochera C. Camacho A. Michaud L. (2017). Prokaryotic assemblages in the maritime Antarctic Lake Limnopolar (Byers Peninsula, South Shetland Islands). *Extremophiles* 21, 947–961. 10.1007/s00792-017-0955-x 28936677

[B68] Pausan M. R. Csorba C. Singer G. Till H. Schöpf V. Santigli E. (2019). Exploring the archaeome: detection of archaeal signatures in the human body. *Front. Microbiol.* 10:2796. 10.3389/fmicb.2019.02796 31866971 PMC6906140

[B69] Porazinska D. L. Fountain A. G. Nylen T. H. Tranter M. Virginia R. A. Wall D. H. (2004). The Biodiversity and biogeochemistry of cryoconite holes from McMurdo Dry Valley glaciers, Antarctica. *Arct. Antarct. Alp. Res.* 36 84–91.

[B70] Porter K. G. Feig Y. S. (1980). The use of DAPI for identifying and counting aquatic microflora 1. *Limnol. Oceanogr.* 25 943–948.

[B71] Quast C. Pruesse E. Yilmaz P. Gerken J. Schweer T. Yarza P. (2013). The SILVA ribosomal RNA gene database project: improved data processing and web-based tools. *Nucleic Acids Res.* 41 590–596. 10.1093/nar/gks1219 23193283 PMC3531112

[B72] Revelle W. (2019). psych: Procedures for Psychological, Psychometric, and Personality Research. Northwestern University, Evanston, Illinois. R package version 1.9.12. Available online at: https://CRAN.R-project.org/package=psych (accessed February 15, 2020).

[B73] Rignot E. Mouginot J. Morlighem M. Seroussi H. Scheuchl B. (2014). Widespread, rapid grounding line retreat of Pine Island, Thwaites, Smith, and Kohler glaciers, West Antarctica, from 1992 to 2011. *Geophys. Res. Lett.* 41 3502–3509. 10.1002/2014GL060140

[B74] Samui G. Antony R. Thamban M. (2018). Chemical characteristics of hydrologically distinct cryoconite holes in coastal Antarctica. *Ann. Glaciol.* 59 69–76. 10.1017/aog.2018.30

[B75] Scheinert M. Ivins E. Dietrich R. Rülke A. (2006). “Vertical Crustal Deformation in Dronning Maud Land, Antarctica: Observation versus Model Prediction,” in *Antarctica*, eds Fütterer D. K. Damaske D. Kleinschmidt G. Miller H. Tessensohn F. (Berlin: Springer).

[B76] Schindelin J. Arganda-Carreras I. Frise E. Kaynig V. Longair M. Pietzsch T. (2012). Fiji: an open-source platform for biological-image analysis. *Nat. Methods* 9 676–682. 10.1038/nmeth.2019 22743772 PMC3855844

[B77] Schwab M. (1998). Reconstruction of the late quaternary climatic and environmental history of the Schirmacher Oasis and the Wohlthat Massif (East Antarctica). *Rep. Polar Res.* 293 1–128. 10.1016/j.quaint.2010.11.025

[B78] Smith H. J. Schmit A. Foster R. Littman S. Kuypers M. M. Foreman C. M. (2016). Biofilms on glacial surfaces: hotspots for biological activity. *NPJ Biofilms Microbiomes* 2:16008. 10.1038/npjbiofilms.2016.8 28721245 PMC5515272

[B79] Sommers P. Fontenele R. S. Kringen T. Kraberger S. Porazinska D. L. Darcy J. L. (2019). Single-stranded DNA viruses in antarctic cryoconite holes. *Viruses* 11:1022. 10.3390/v11111022 PMC6893807 31689942

[B80] Takeuchi N. Kohshima S. Seko K. (2001). Structure, Formation, and Darkening Process of Albedo-reducing Material (Cryoconite) on a Himalayan Glacier: a Granular Algal Mat Growing on the Glacier. *Arctic Antarct. Alp. Res.* 33 115–122. 10.1080/15230430.2001.12003413

[B81] Tang C. Madigan M. T. Lanoil B. (2013). Bacterial and archaeal diversity in sediments of west Lake Bonney, McMurdo Dry Valleys, Antarctica. *Appl. Environ. Microbiol.* 79 1034–1038. 10.1128/AEM.02336-12 23183970 PMC3568539

[B82] Telling J. Anesio A. M. Tranter M. Fountain A. G. Nylen T. Hawkings J. (2014). Spring thaw ionic pulses boost nutrient availability and microbial growth in entombed Antarctic Dry Valley cryoconite holes. *Front. Microbiol.* 5:694. 10.3389/fmicb.2014.00694 25566210 PMC4263180

[B83] Tikhonov M. Leach R. W. Wingreen N. S. (2015). Interpreting 16S metagenomic data without clustering to achieve sub-OTU resolution. *ISME J.* 9 68–80. 10.1038/ismej.2014.117 25012900 PMC4274427

[B84] Uetake J. Nagatsuka N. Onuma Y. Takeuchi N. Motoyama H. Aoki T. (2019). Bacterial community changes with granule size in cryoconite and their susceptibility to exogenous nutrients on NW Greenland glaciers. *FEMS Microbiol. Ecol.* 95:fiz075. 10.1093/femsec/fiz075 31132102

[B85] Vincent W. F. (2000). “Cyanobacterial dominance in the polar regions,” in *The Ecology of Cyanobacteria*, eds Whitton B. A. Potts M. (Dordrecht: Springer), 321–340.

[B86] Vincent W. F. Gibson J. A. E. Pienitz R. Villeneuve V. Broady P. A. Hamilton P. B. (2000). Ice shelf microbial ecosystems in the high Arctic and implications for life on Snowball Earth. *Naturwissenschaften* 87 137–141. 10798200 10.1007/s001140050692

[B87] Vogler P. (1966). Zur Analytik kondensierter Phosphate und organischer Phosphate bei limnologischen Untersuchungen. *Int. Revue ges. Hydrobiol. Hydrogr.* 51 775–785. 10.1002/iroh.19660510507

[B88] Wand U. Perlt J. (1999). Glacial boulders ‘floating’ on the ice cover of Lake Untersee, East Antarctica. *Antarct. Sci.* 11 256–260. 10.1017/S0954102099000310

[B89] Wand U. Samarkin V. A. Nitzsche H. M. Hubberten H. W. (2006). Biogeochemistry of methane in the permanently ice-covered Lake Untersee, central Dronning Maud Land, East Antarctica. *Limnol. Oceanogr.* 51 1180–1194.

[B90] Webster-Brown J. Gall M. Gibson J. Wood S. Hawes I. (2010). The biogeochemistry of meltwater habitats in the Darwin Glacier region (80oS), Victoria Land, Antarctica. *Antarct. Sci.* 22 646–661. 10.1017/S0954102010000787

[B91] Webster-Brown J. G. Hawes I. Jungblut A. D. Wood S. A. Christenson H. K. (2015). The effects of entombment on water chemistry and bacterial assemblages in closed cryoconite holes on Antarctic glaciers. *FEMS Microbiol. Ecol.* 91:fiv144. 10.1093/femsec/fiv144 26572547

[B92] Weisleitner K. Hunger L. Kohstall C. Frisch A. Storrie-Lombardi M. C. Sattler B. (2019a). Laser-Induced Fluorescence Emission (LIFE) as Novel Non-Invasive Tool for In-Situ Measurements of Biomarkers in Cryospheric Habitats. *J. Vis. Exp.* 152:e60447. 10.3791/60447 31710034

[B93] Weisleitner K. Perras A. K. Moissl-Eichinger C. Andersen D. T. Sattler B. (2019b). Source environments of the microbiome in perennially ice-covered Lake Untersee, Antarctica. *Front. Microbiol.* 10:1019. 10.3389/fmicb.2019.01019 31134036 PMC6524460

[B94] Wharton R. A. McKay C. P. Simmons G. M. Parker B. C. (1985). Cryoconite holes on glaciers. *Bioscience* 35 499–503. 10.2307/1309818 11539025

[B95] Wharton R. A. Vinyard W. C. Parker B. C. Simmons G. M. Seaburg K. G. (1981). Algae in cryoconite holes on Canada Glacier in Southern Victorialand, Antarctica. *Phycologia* 20 208–211. 10.2216/i0031-8884-20-2-208.1

[B96] Wickham H. (2011). ggplot2. *WIREs Comp. Stat.* 3 180–185. 10.1002/wics.147

[B97] Yergeau E. Newsham K. K. Pearce D. A. Kowalchuk G. A. (2007). Patterns of bacterial diversity across a range of Antarctic terrestrial habitats. *Environ. Microbiol.* 9 2670–2682. 17922752 10.1111/j.1462-2920.2007.01379.x

[B98] Zdanowski M. K. Bogdanowicz A. Gawor J. Gromadka R. Wolicka D. Grzesiak J. (2017). Enrichment of cryoconite hole anaerobes: implications for the subglacial microbiome. *Microb. Ecol.* 73 532–538. 10.1007/s00248-016-0886-6 27822618 PMC5348551

[B99] Zeglin L. H. Dahm C. N. Barrett J. E. Gooseff M. N. Fitpatrick S. K. Takacs-Vesbach C. D. (2011). Bacterial community structure along moisture gradients in the parafluvial sediments of two ephemeral desert streams. *Microb. Ecol.* 61, 543–556. 10.1007/s00248-010-9782-7 21153024

