# Supplementary material for: Cryoconite Hole Location in East-Antarctic Untersee Oasis Shapes Physical and Biological Diversity
Source: Front Microbiol. 2020 Jun 3;11:1165. doi: 10.3389/fmicb.2020.01165 (PMC7284004; doi:10.3389/fmicb.2020.01165)
Supplement: Supplementary file 1 [file Data_Sheet_1.docx]

Supplementary Material

Cryoconite Hole Location in East-Antarctic Untersee Oasis Shapes Physical and Biological Diversity

Klemens Weisleitner Alexandra Kristin Perras, Seraphin Hubert Unterberger, Christine Moissl-Eichinger, Dale T. Andersen, Birgit Sattler*

*** Correspondence:** Birgit Sattler: [Birgit.Sattler@uibk.ac.at](mailto:Birgit.Sattler@uibk.ac.at)

**8 Supplementary Figures and 3 Supplementary Tables**


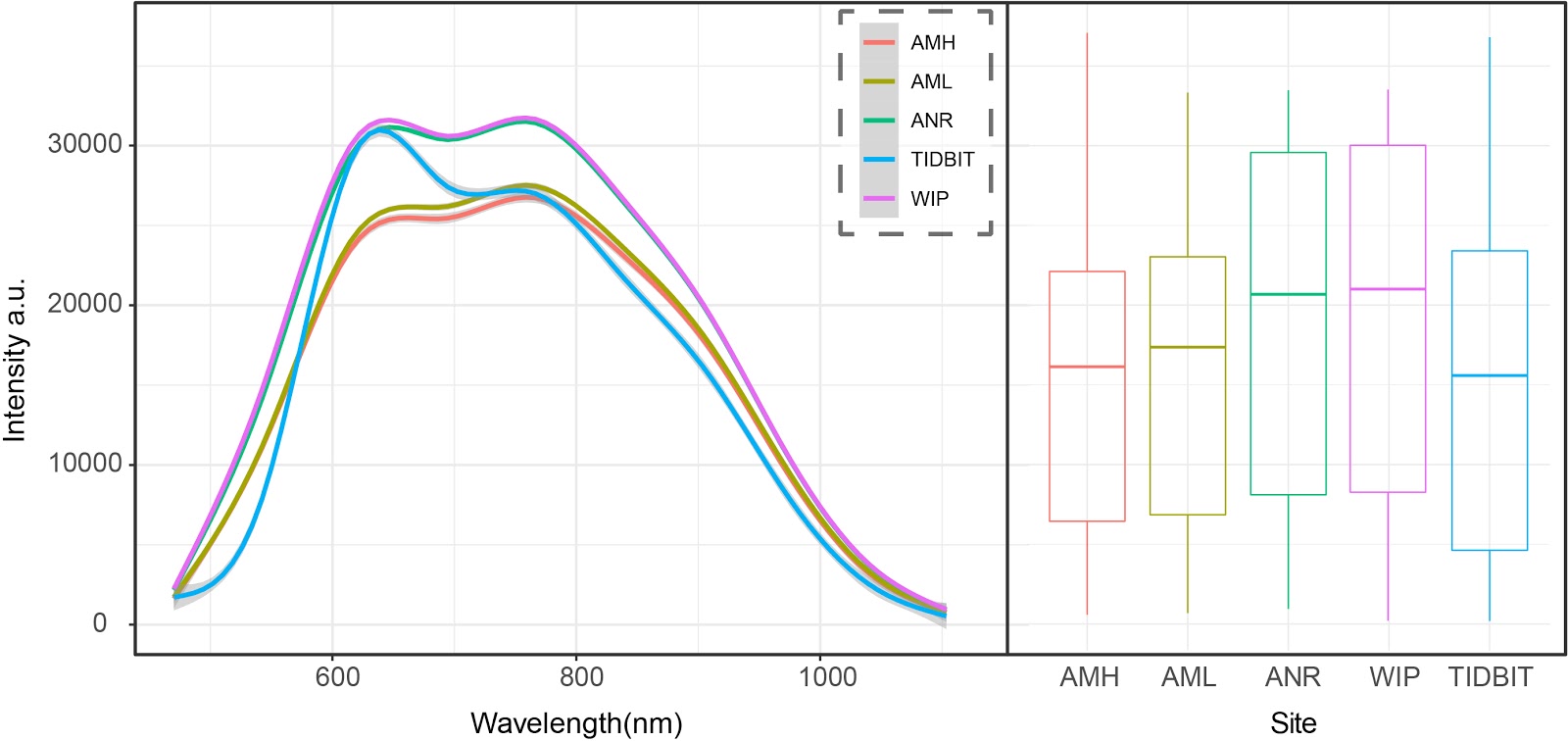


**Supplementary Figure 1:** Comparison of relative brightness between CH samples (AMH, AML, ANR, WIP) and the Tidbit temperature loggers. The samples and loggers were illuminated with a white LED and a tungsten filament. Reflected light was measured with a custom-built device using an Ibsen OEM FHT-315 spectrometer with an integration time of 600 ms in a range of 470 nm and 1100 nm under controlled laboratory conditions. Left: Averaged spectra from each site in comparison with the Tidbit logger. Right: Boxplots of relative brightness values. The median brightness of all CHs was 17,448.93. The median Tidbit brightness was slightly higher (18,603.06) but within the range of the CH samples.


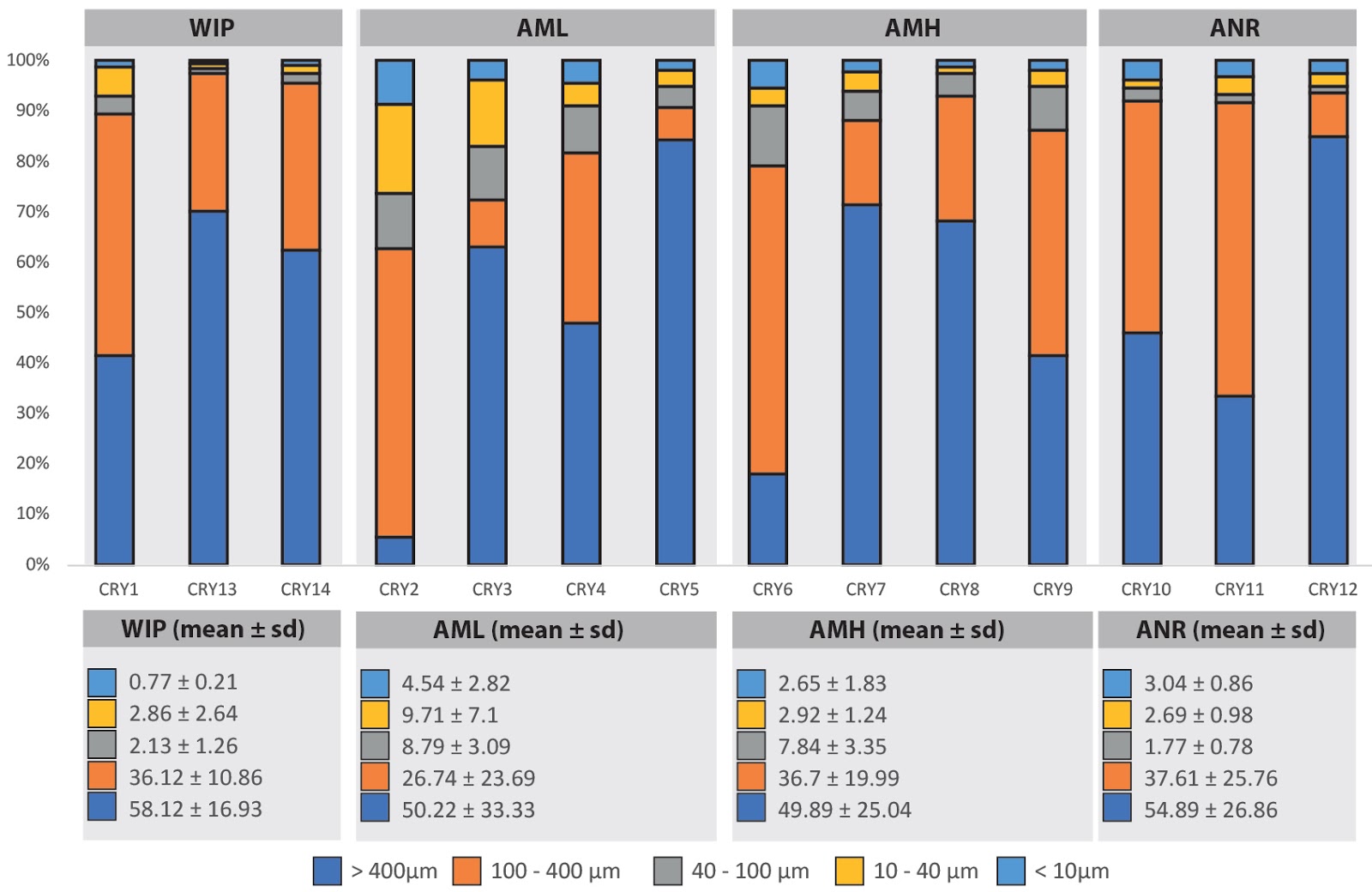


**Supplementary Figure 2:** Gravimetric particle size distribution of all samples (top) and mean values (± sd) from each site (bottom).


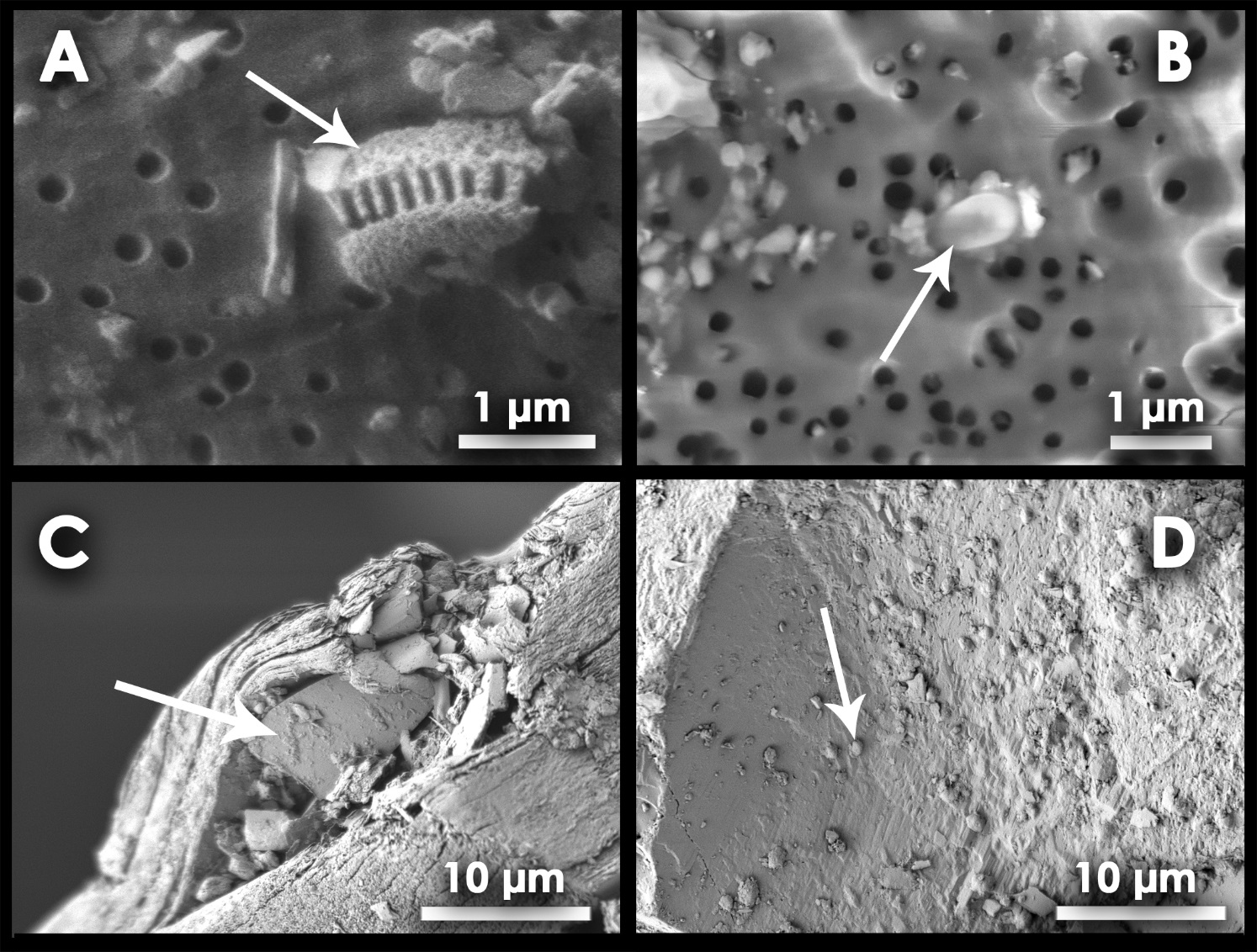


**Supplementary Figure 3:** Representative SEM images of cryoconite samples on a 0.2 µm polycarbonate filter (upper row) and after detaching cells from the sediment (bottom). A: Diatom frustule. B: Prokaryotic cell with mineral particles attached to it. C: A mineral stuck between layers of biotite; D: Prokaryotic cells in a matrix that covers the mineral surface. Key features in each image are highlighted with an arrow.


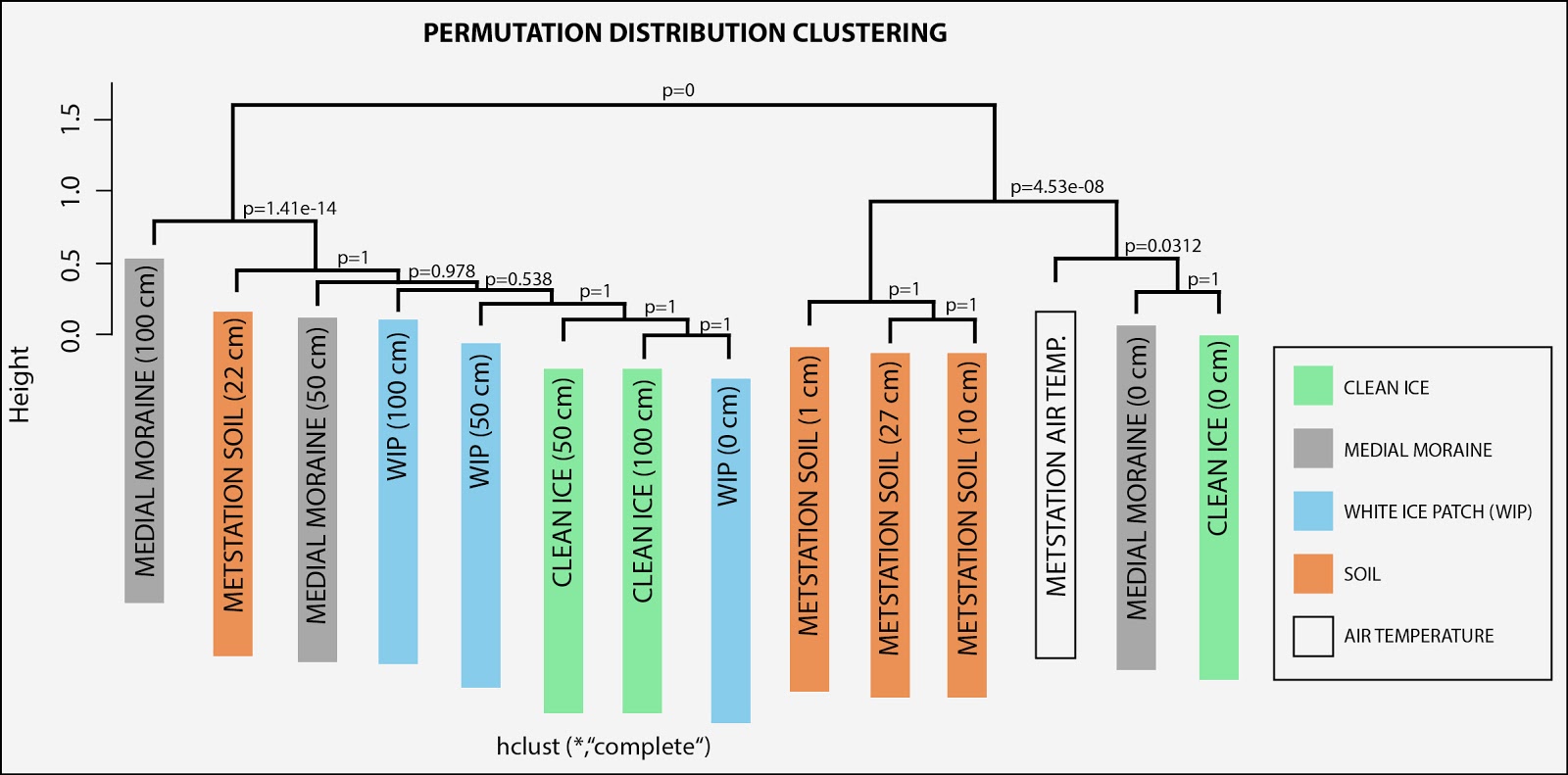


**Supplementary Figure 4**: Permutation clustering of temperature recorded in ice, soils and air.


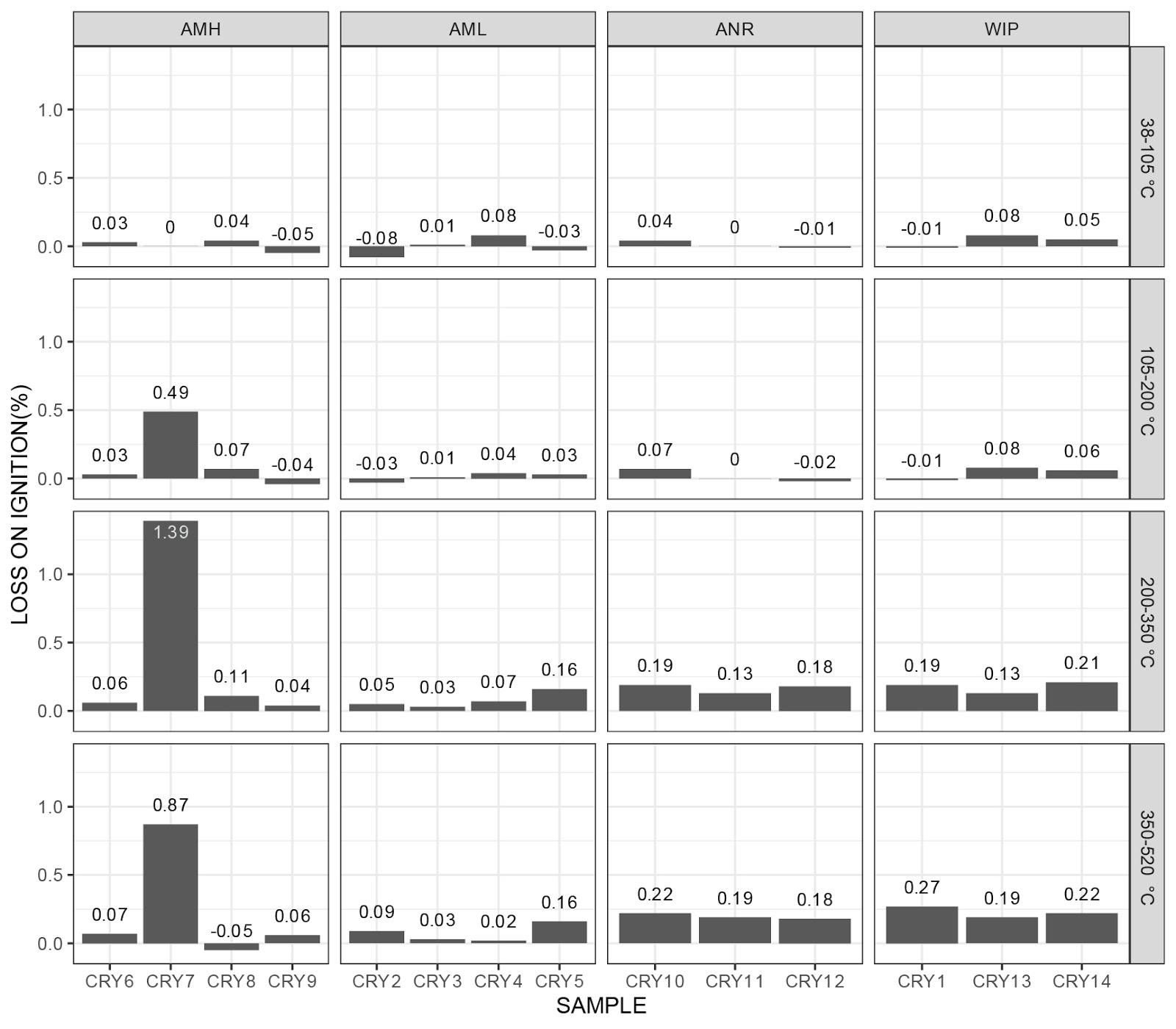


**Supplementary Figure 5:** Organic matter contents in cryoconite. Labile and stable organic matter are represented by the 200 - 350 °C and the 350 - 520 °C fractions, respectively. Sample CRY-7 was identified as an outlier most that was most likely caused by the presence of hydrogen-bonded water in the mineral laumontite.


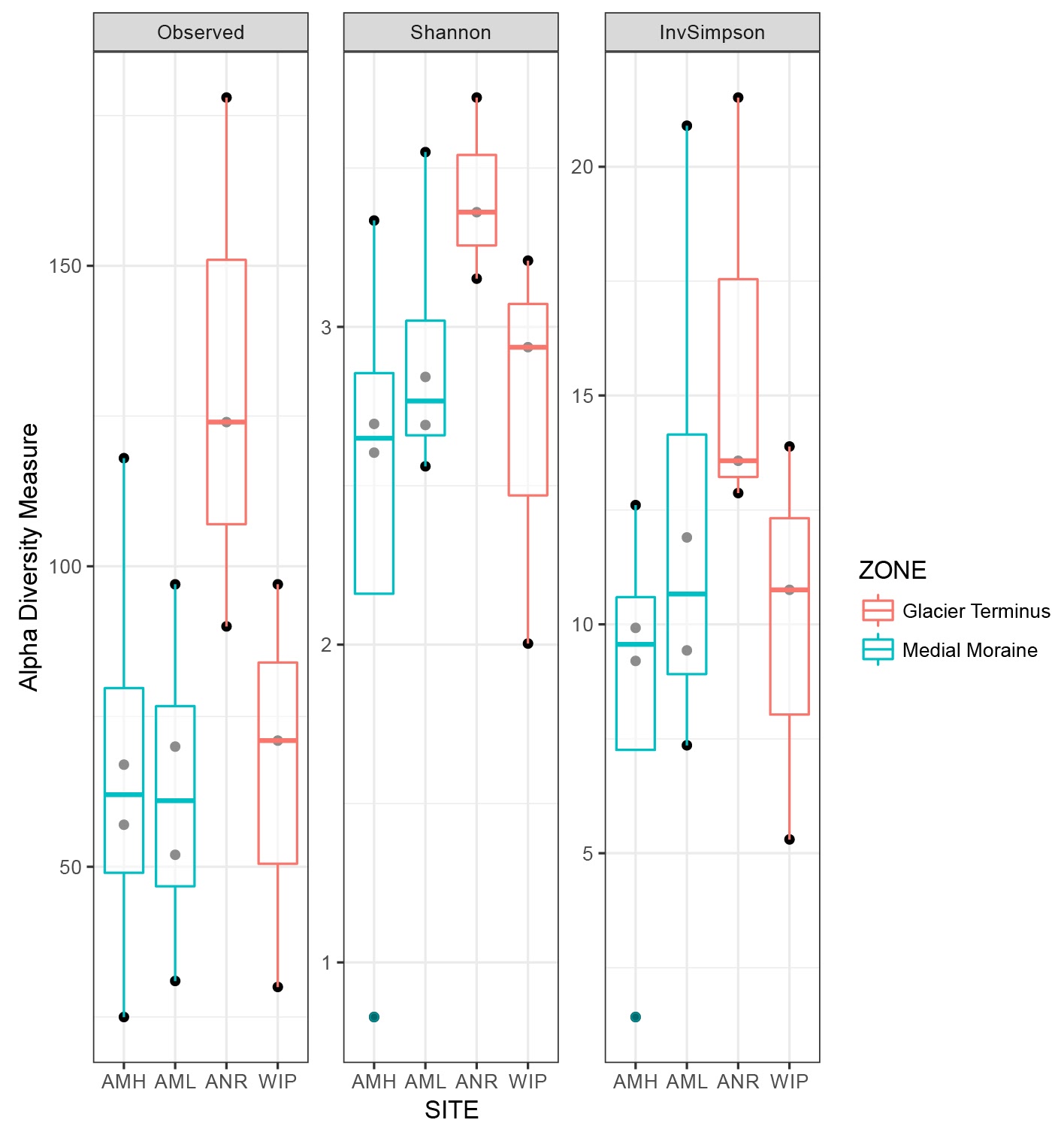


Supplementary Figure 6: Site comparison of alpha diversity measures.





**Supplementary Figure 7:** Bacterial RSVs (top) and archaeal RSVs (bottom) exceeding the ≥ 0.1% threshold according to their occurrence from 1 (i.e. occurrence at only one site) to 4 sites (i.e. core microbiome).


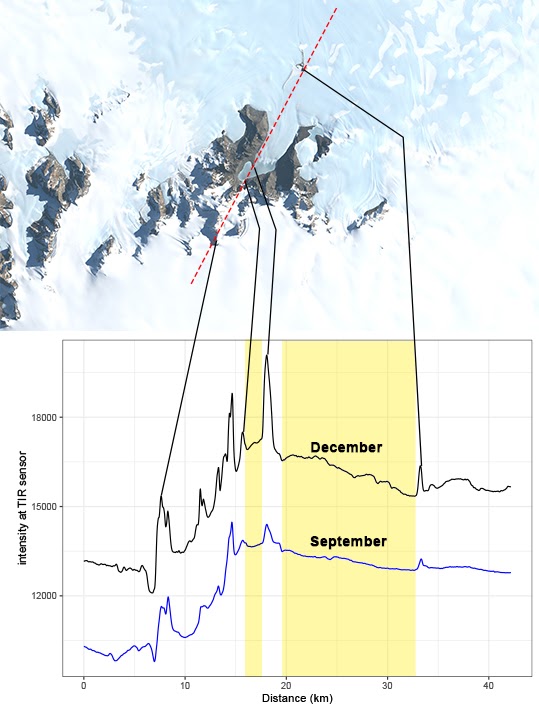


**Supplementary Figure 8:** Relative thermal emission captured by the Landsat 8 thermal infrared imager. Both thermal bands (band 10 (10.6 - 11.2 µm), band 11 (11.5 - 12.5 µm) were averaged prior analysis. The red line indicates the measured path (average of 4 pixels (400 m) along 42.18 km).

**Supplementary Table 1:** Accession numbers and site information for all samples. AML = Anuchin Medial moraine LOW, AMH = Anuchin Medial moraine High, ANR = Anuchin North of Ridge, WIP = White Ice Patch.

| Sample | target | Accession number | Site |
| --- | --- | --- | --- |
| CRY 1 | Archaea | SRS3297449 | WIP |
| CRY 2 | Archaea | SRS3297448 | AML |
| CRY 3 | Archaea | SRS3297455 | AML |
| CRY 4 | Archaea | SRS3297454 | AML |
| CRY 5 | Archaea | SRS3297453 | AML |
| CRY 6 | Archaea | SRS3297452 | AMH |
| CRY 7 | Archaea | SRS3297447 | AMH |
| CRY 8 | Archaea | SRS3297446 | AMH |
| CRY 9 | Archaea | SRS3297547 | AMH |
| CRY 10 | Archaea | SRS3297549 | ANR |
| CRY 11 | Archaea | SRS3297546 | ANR |
| CRY 12 | Archaea | SRS3297548 | ANR |
| CRY 13 | Archaea | SRS3297545 | WIP |
| CRY 14 | Archaea | SRS3297544 | WIP |
| CRY 1 | Bacteria | SRS3297511 | WIP |
| CRY 2 | Bacteria | SRS3297510 | AML |
| CRY 3 | Bacteria | SRS3297509 | AML |
| CRY 4 | Bacteria | SRS3297507 | AML |
| CRY 5 | Bacteria | SRS3297516 | AML |
| CRY 6 | Bacteria | SRS3297514 | AMH |
| CRY 7 | Bacteria | SRS3297513 | AMH |
| CRY 8 | Bacteria | SRS3297512 | AMH |
| CRY 9 | Bacteria | SRS3297508 | AMH |
| CRY 10 | Bacteria | SRS3297506 | ANR |
| CRY 11 | Bacteria | SRS3297505 | ANR |
| CRY 12 | Bacteria | SRS3297504 | ANR |
| CRY 13 | Bacteria | SRS3297503 | WIP |
| CRY 14 | Bacteria | SRS3297502 | WIP |

**Supplementary Table 2:** Permutational Multivariate Analysis of Variance Using Distance Matrices - Variables explaining variation in community structure.

Call:

adonis(formula = Bray-Curtis_distancematrix ~ Depth + OM + TC + TN + TP + BCP + Diameter + Abundance + Mineral_diversity + Particles<10µm + Particles10-40µm + Particles40-100µm, data = sampledf, strata = Site).

| Explanatory variable | F-model | Explained variance (%) | P value |
| --- | --- | --- | --- |
| Depth | 8.3584 | 20.518 | **0.001** |
| Total OM | 4.2664 | 10.473 | **0.003** |
| TC | 2.1559 | 5.292 | 0.069 |
| TN | 0.7836 | 3.993 | 0.158 |
| TP | 3.3386 | 8.195 | **0.016** |
| BCP | 2.5923 | 6.363 | **0.037** |
| Diameter | 1.8508 | 4.543 | 0.110 |
| Abundance | 2.6922 | 6.609 | **0.027** |
| Mineral diversity | 4.9051 | 12.041 | **0.002** |
| Particles  < 10 µm  10-40 µm  40-100 µm | 2.5961  2.3762  2.9783 | 6.373  5.833  7.311 | 0.307  0.401  **0.015** |

**Supplementary Table 3:** Differential abundance analysis output (DESEQ 2) showing significantly different abundant RSVs between the medial moraine and glacier terminus.

| #RSV | log2Fold  Change | Padj. | Phlyum | Class | Order | Family | Genus |
| --- | --- | --- | --- | --- | --- | --- | --- |
| 15 | 12.38 | 1.32*10^-9^ | Proteobacteria | Gammaproteobacteria | Xanthomonadales | Xanthomonadaceae | Silanimonas |
| 30 | 11.31 | 1.61*10^-05^ | Cyanobacteria | Cyanobacteria | SubsectionIII | FamilyI | Leptolyngbya |
| 46 | -8.17 | 7.71*10^-03^ | Actinobacteria | Actinobacteria | Micrococcales | Microbacteriaceae | Salinibacterium |
| 53 | 9.83 | 1.42*10^-05^ | Bacteroidetes | Flavobacteriia | Flavobacteriales | Flavobacteriaceae | Flavobacterium |
| 63 | 7.74 | 1.89*10^-03^ | Bacteroidetes | Cytophagia | Cytophagales | Cyclobacteriaceae | Algoriphagus |
| 70 | 8.75 | 1.89*10^-03^ | Proteobacteria | Gammaproteobacteria | Xanthomonadales | Xanthomonadaceae | Lysobacter |
| 76 | -8.65 | 3.67*10^-03^ | Cyanobacteria | Cyanobacteria | SubsectionIII | FamilyI | Leptolyngbya |
| 93 | 8.12 | 2.38*10^-04^ | Bacteroidetes | Flavobacteriia | Flavobacteriales | Flavobacteriaceae | Flavobacterium |
| 108 | -8.70 | 1.89*10^-03^ | Bacteroidetes | Cytophagia | Cytophagales | Cytophagaceae | Spirosoma |
| 111 | 7.19 | 5.18*10^-03^ | Actinobacteria | Thermoleophilia | Solirubrobacterales | 480-2 | not assigned |
| 112 | 6.56 | 8.57*10^-03^ | Proteobacteria | Betaproteobacteria | Burkholderiales | Comamonadaceae | Hydrogenophaga |
| 166 | 8.07 | 1.89*10^-03^ | Proteobacteria | Alphaproteobacteria | Sphingomonadales | Sphingomonadaceae | Sphingopyxis |
| 172 | 7.01 | 5.55*10^-03^ | Planctomycetes | Planctomycetacia | Planctomycetales | Planctomycetaceae | not assigned |
| 200 | 7.08 | 5.55*10^-03^ | Armatimonadetes | not assigned | not assigned | not assigned | not assigned |
| 213 | 8.80 | 3.67*10^-03^ | Proteobacteria | Gammaproteobacteria | Xanthomonadales | Xanthomonadaceae | Rhodanobacter |
| 249 | 9.45 | 1.42*10^-05^ | Bacteroidetes | Flavobacteriia | Flavobacteriales | Flavobacteriaceae | Flavobacterium |
| 257 | -7.51 | 6.79*10^-03^ | Proteobacteria | Alphaproteobacteria | Sphingomonadales | Sphingomonadaceae | Sphingomonas |
| 258 | 9.37 | 1.48*10^-05^ | Bacteroidetes | Sphingobacteriia | Sphingobacteriales | Sphingobacteriaceae | Pedobacter |
| 310 | 7.09 | 5.55*10-03 | Saccharibacteria | not assigned | not assigned | not assigned | not assigned |
| 163 | 10.62 | 1.48*10-05 | Bacteroidetes | Cytophagia | Cytophagales | Cyclobacteriaceae | Algoriphagus |
| 181 | 10.79 | 2.65*10-07 | Proteobacteria | Betaproteobacteria | Burkholderiales | Comamonadaceae | Rhizobacter |
| 188 | 8.34 | 2.83*10-03 | Proteobacteria | Betaproteobacteria | Burkholderiales | Comamonadaceae | Hydrogenophaga |
| 195 | 7.31 | 3.67*10-03 | Planctomycetes | Planctomycetacia | Planctomycetales | Planctomycetaceae | Pirellula |
| 286 | 7.21 | 8.71*10-03 | Proteobacteria | Alphaproteobacteria | Rhodospirillales | Acetobacteraceae | Roseomonas |
| 287 | 7.61 | 2.52*10-03 | Actinobacteria | Actinobacteria | Micrococcales | Intrasporangiaceae | Aquipuribacter |
| 309 | 7.32 | 1.63*10-03 | Proteobacteria | Alphaproteobacteria | Rhodobacterales | Rhodobacteraceae | Rhodobacter |
